# XBP1 signalling is essential for alleviating mutant protein aggregation in ER-stress related skeletal disease

**DOI:** 10.1371/journal.pgen.1008215

**Published:** 2019-07-01

**Authors:** Katarzyna A. Piróg, Ella P. Dennis, Claire L. Hartley, Robert M. Jackson, Jamie Soul, Jean-Marc Schwartz, John F. Bateman, Raymond P. Boot-Handford, Michael D. Briggs

**Affiliations:** 1 Institute of Genetic Medicine, Newcastle University, Newcastle, United Kingdom; 2 Wellcome Trust Centre for Cell Matrix Research, University of Manchester, Manchester, United Kingdom; 3 Murdoch Children's Research Institute, Parkville, Victoria, Australia; University of Glasgow, UNITED KINGDOM

## Abstract

The unfolded protein response (UPR) is a conserved cellular response to the accumulation of proteinaceous material in endoplasmic reticulum (ER), active both in health and disease to alleviate cellular stress and improve protein folding. Multiple epiphyseal dysplasia (EDM5) is a genetic skeletal condition and a classic example of an intracellular protein aggregation disease, whereby mutant matrilin-3 forms large insoluble aggregates in the ER lumen, resulting in a specific ‘disease signature’ of increased expression of chaperones and foldases, and alternative splicing of the UPR effector XBP1. Matrilin-3 is expressed exclusively by chondrocytes thereby making EDM5 a perfect model system to study the role of protein aggregation in disease. In order to dissect the role of XBP1 signalling in aggregation-related conditions we crossed a p.V194D *Matn3* knock-in mouse model of EDM5 with a mouse line carrying a cartilage specific deletion of XBP1 and analysed the resulting phenotype. Interestingly, the growth of mice carrying the *Matn3* p.V194D mutation compounded with the cartilage specific deletion of XBP1 was severely retarded. Further phenotyping revealed increased intracellular retention of amyloid-like aggregates of mutant matrilin-3 coupled with dramatically decreased cell proliferation and increased apoptosis, suggesting a role of XBP1 signalling in protein accumulation and/or degradation. Transcriptomic analysis of chondrocytes extracted from wild type, EDM5, *Xbp1*-null and compound mutant lines revealed that the alternative splicing of *Xbp1* is crucial in modulating levels of protein aggregation. Moreover, through detailed transcriptomic comparison with a model of metaphyseal chondrodysplasia type Schmid (MCDS), an UPR-related skeletal condition in which XBP1 was removed without overt consequences, we show for the first time that the differentiation-state of cells within the cartilage growth plate influences the UPR resulting from retention of a misfolded mutant protein and postulate that modulation of XBP1 signalling pathway presents a therapeutic target for aggregation related conditions in cells undergoing proliferation.

## Author summary

A significant proportion of genetic skeletal diseases result from misfolding of structural proteins within the endoplasmic reticulum (ER) of cartilage cells (chondrocytes). Interestingly, these diseases often share transcriptomic changes with non-skeletal conditions such as Alzheimer’s disease and diabetes and include changes in the unfolded protein response (UPR). UPR signals are conveyed through three effector molecules, IRE1, ATF6 and PERK, understanding of which is of utmost importance for the development of novel therapeutic approaches. IRE1 is the most conserved UPR pathway, with XBP1 as its main effector. Interestingly, in our study we show that chondrocyte response to ER stress is highly modulated by the differentiation state of the cell. We have deleted the IRE1/XBP1 pathway activity in the chondrocytes of a mouse model of multiple epiphyseal dysplasia (MED), expressing misfolding matrilin-3 predominantly in proliferating chondrocytes, and in metaphyseal chondrodysplasia type Schmid (MCDS), resulting from mutations in type X collagen, expressed by the hypertrophic chondrocytes. Here, using deep phenotyping and extensive transcriptomic analysis we demonstrate that whilst the IRE1/XBP1 pathway is redundant in hypertrophic chondrocyte pathology, it is in fact essential for UPR responses in proliferating cells, making this highly conserved pathway an attractive therapeutic target for a broad range of UPR related conditions.

## Introduction

The unfolded protein response (UPR) is one of the canonical cellular stress pathways that is triggered by unfolded proteins accumulating in the ER lumen. The pathway is active in both health and disease and many secretory cells have a highly active UPR to allow a greater secretory output [1]. Over the recent years, the UPR triggered by ER-stress has been recognised as a crucial component in the pathobiology of many human diseases, including neurodegenerative conditions, diabetes and numerous musculoskeletal phenotypes [2–6]. However, the specific role of the UPR in the context of human disease is still being determined and it is hoped that it will offer attractive therapeutic targets and avenues in the future.

The canonical UPR response is initiated when the chaperone protein BiP dissociates from its three membrane bound receptors, PERK, ATF6 and IRE1 [7]. This dissociation is triggered by the exposed hydrophobic residues of misfolded proteins in the ER lumen and the UPR then proceeds along the three signalling pathways, which are further modulated by the levels and duration of the stress [8]. Furthermore, these branches cross talk and signal in conjunction with, or modulate other important mechanisms such as inflammation and autophagy, thereby offering the cell a robust machinery to counteract protein misfolding and oxidative stress. Following dissociation of BiP, PERK dimerises and autophosphorylates, which in turn triggers the phosphorylation of elongation factor eIF2ɑ. This leads to attenuation of general protein translation allowing the cell to recover from an abnormal protein load [9]. However, several proteins escape this translational block, including ATF4, a downstream effector of PERK, which can trigger ER-stress related apoptosis via CHOP (or DDIT3 [10, 11]. Following release from BiP, ATF6 translocates to the Golgi apparatus where it is cleaved, releasing an active transcription factor. ATF6 signalling then leads to an upregulation of chaperones and *XBP1* expression, but it can also trigger apoptosis via CHOP mediated signalling [12]. The third transmembrane sensor is IRE1, which dimerises and autophosphorylates upon the dissociation of BiP. In the active form IRE1 can induce the alternative splicing of *XBP1*, producing an active transcription factor which upregulates chaperone genes and genes responsible for ER-associated degradation of accumulated proteins (ERAD) [13–15]. It is therefore not surprising that the XBP1 branch of the UPR pathway has been implicated in many protein aggregation diseases including Alzheimers [16, 17], Huntington’s disease [18, 19], type II diabetes [20] and several skeletal conditions [5, 7, 12, 21] amongst others.

Multiple epiphyseal dysplasia (MED) is predominantly an autosomal dominant skeletal dysplasia characterised by disproportionate short-limbed dwarfism and early onset joint degeneration [22]. MED results from dominant-negative mutations in three structural proteins of the cartilage extracellular matrix (ECM); cartilage oligomeric matrix protein (COMP), matrilin-3 and type IX collagen, which interact with each other in the ECM [23]. Matrilin-3 is a tetrameric bridging molecule that regulates collagen fibrillogenesis [24] and each monomer consists of a single von Willebrand factor A like domain (A-domain), four EGF-like repeats and an oligomerisation domain. MED-causing mutations (EDM5; OMIM #607078) are located exclusively in the disulphide bond stabilised A-domain and result in misfolding and retention of the mutant protein in the ER lumen. A classical UPR is activated with an upregulation of generic chaperones as well as a more specific cocktail of disulphide isomerases such as CRELD2, PDIA1, PDIA3 and PDIA6 [5]. However, the mutant protein is prone to aggregation, forming large insoluble non-native disulphide bonded aggregates in the ER that contain a high percentage of ß-sheet folds [5, 25], suggesting a propensity to form amyloid-like deposits upon misfolding [26] that appear resistant to degradation. This in turns leads to the dysregulation of chondrocyte apoptosis, a decrease in chondrocyte proliferation and consequently reduced bone growth [27, 28].

We have previously demonstrated that chondrocytes from a mouse model of EDM5 with a p.V194D mutation in *Matn3* exhibited a specific upregulation of genes in the XBP1 branch of the UPR [5, 29, 30]. A similar upregulation of XBP1 signalling was seen in the *Col10a1* N617K model of metaphyseal chondrodysplasia type Schmid (MCDS), but not in an allelic series of pseudoachondroplasia (PSACH) causing *Comp* mutations, indicating gene product specificity of this arm of the UPR [6, 31, 32]. Therefore, in order to further understand the role of XBP1 signalling in the protein aggregation and disease pathology of *Matn3*-related MED we crossed a p.V194D knock-in mouse model of EDM5 with a mouse line carrying a cartilage specific deletion of *Xbp1* and analysed in-depth the resulting phenotype.

## Results

### Engineering the chondrocyte specific knock out of *Xbp1* in the EDM5 mouse model

**Fig 1:**
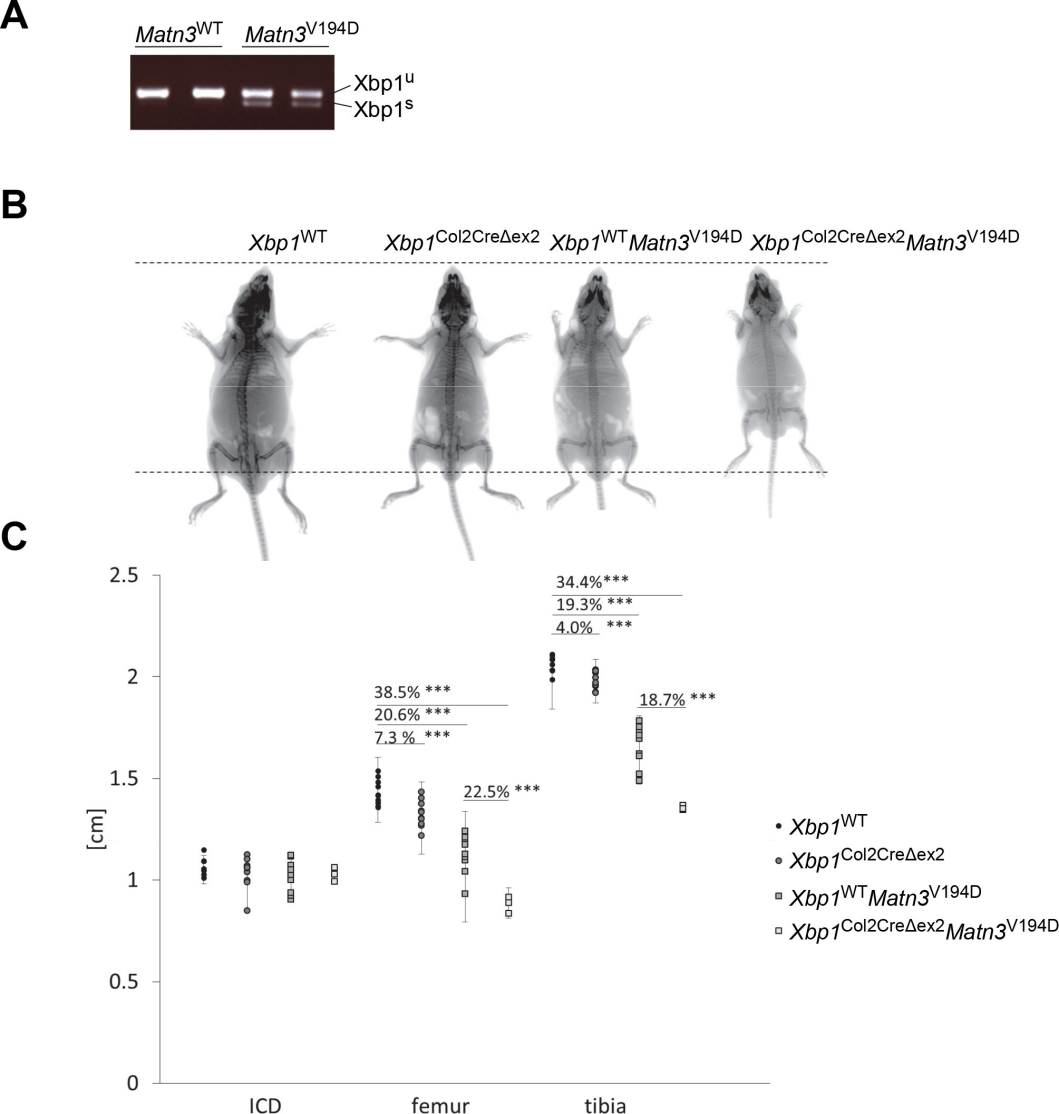
(A) Mutation in MATN3 induced an UPR response and alternative splicing of *Xbp1*. (B) *Xbp1*^Col2CreΔex2^ mice presented with a mild chondrodysplasia and were shorter than their wild type littermates. *Xbp1*^WT^*Matn3*^V194D^ mice presented with typical EDM5 short limbed dwarfism. The skeletal phenotype of *Xbp1*^Col2CreΔex2^ *Matn3*^V194D^ was very severe, with short limbs, constricted ribcages and a pronounced hip dysplasia. (C) Bone measurements using Xray analysis at 3, 6 and 9 weeks of age show disruption of endochondral ossification (tibia, femur), but not the intramembranous ossification (inner canthal distance, ICD) in the *Xbp1*^Col2CreΔex2^, *Xbp1*^WT^ *Matn3*^V194D^ and *Xbp1*^Col2CreΔex2^ *Matn3*^V194D^ mice (n≥5, Student t-test). Key: Xbp1^u^ - unspliced Xbp1, Xbp1^s^ –spliced Xbp1, *** P<0.001.

We have previously generated a mouse model of EDM5 (p.V194D in *Matn3* and referred to as *Xbp1*^WT^ *Matn3*^V194D^ in this paper, [5]). Interestingly, RT-PCR and sequencing of cDNA derived from wild type (*Xbp1*^WT^ *Matn3*^WT^) and homozygous mutant (*Xbp1*^WT^ *Matn3*^V194D^) cartilage dissected from 5-day-old mice revealed non-conventional splicing of *Xbp1* in *Xbp1*^WT^ *Matn3*^V194D^ chondrocytes (Fig 1A). The *Xbp1*^WT^ *Matn3*^V194D^ mouse line was therefore crossed with a mouse line in which *Xbp1* had been rendered inactive in chondrocytes through the *Col2a1*-Cre/*loxP*-mediated deletion of exon 2 (*Xbp1*^Col2CreΔex2^, [21, 31]), in order to study the role of XBP1 signalling in EDM5. This breeding strategy generated the *Xbp1*^Col2CreΔex2^ *Matn3*^V194D^ mouse line. *Xbp1*^Col2CreΔex2^ *Matn3*^V194D^ mice were viable and fertile; however, mice homozygous for both mutant alleles had breeding and survival complications due to their dramatically reduced size, breathing difficulties and narrower birth canals.

### The loss of XBP1 from cartilage leads to an exacerbation of the EDM5 phenotype

Bone measurements were used to determine the effect of XBP1 deletion on endochondral (tibia and femur lengths) and intramembranous (inner canthal distance) ossification of the *Xbp1*^WT^ *Matn3*^V194D^ mouse model. The *Xbp1*^Col2CreΔex2^ mice were slightly shorter than their wild type littermates, as previously reported, indicating a role for Xbp1 in normal skeletal development [21]. *Xbp1*^WT^ *Matn3*^V194D^ mice were shorter than both wild type mice and *Xbp1*^Col2CreΔex2^ mice with a comparable genetic background [33]. Unsurprisingly, the *Xbp1*^Col2CreΔex2^ *Matn3*^V194D^ mice had a more pronounced short-limbed dwarfism than that previously reported for the *Xbp1*^WT^ *Matn3*^V194D^ mice, signifying a crucial protective role of the XBP1 branch of UPR in EDM5 pathology (Fig 1B and 1C). Paradoxically, this is in direct contrast to the minor role for XBP1 signalling proposed in the recent study of the *Col10a*^N617K^ model of metaphyseal chondrodysplasia type Schmid (MCDS) [31].

The *Xbp1*^Col2CreΔex2^ *Matn3*^V194D^ mice had dramatically shorter long bones (>30% reduction compared to wild type mice and ~20% reduction compared to the *Xbp1*^WT^ *Matn3*^V194D^ mice) and abnormal bell-shaped rib cages, which appeared to hinder their ability to breathe correctly. The inner canthal distance (ICD) was not altered in any of the mice studied indicating that intramembranous ossification was not affected. Over time there was severe truncation and rotation of the limb, abnormal bending of the long bones and severe constriction of the rib cages in *Xbp1*^Col2CreΔex2^ *Matn3*^V194D^ mice (S1 Fig).

### Ablation of XBP1 dramatically altered *Matn3*^V194D^ cartilage growth plate morphology

**Fig 2:**
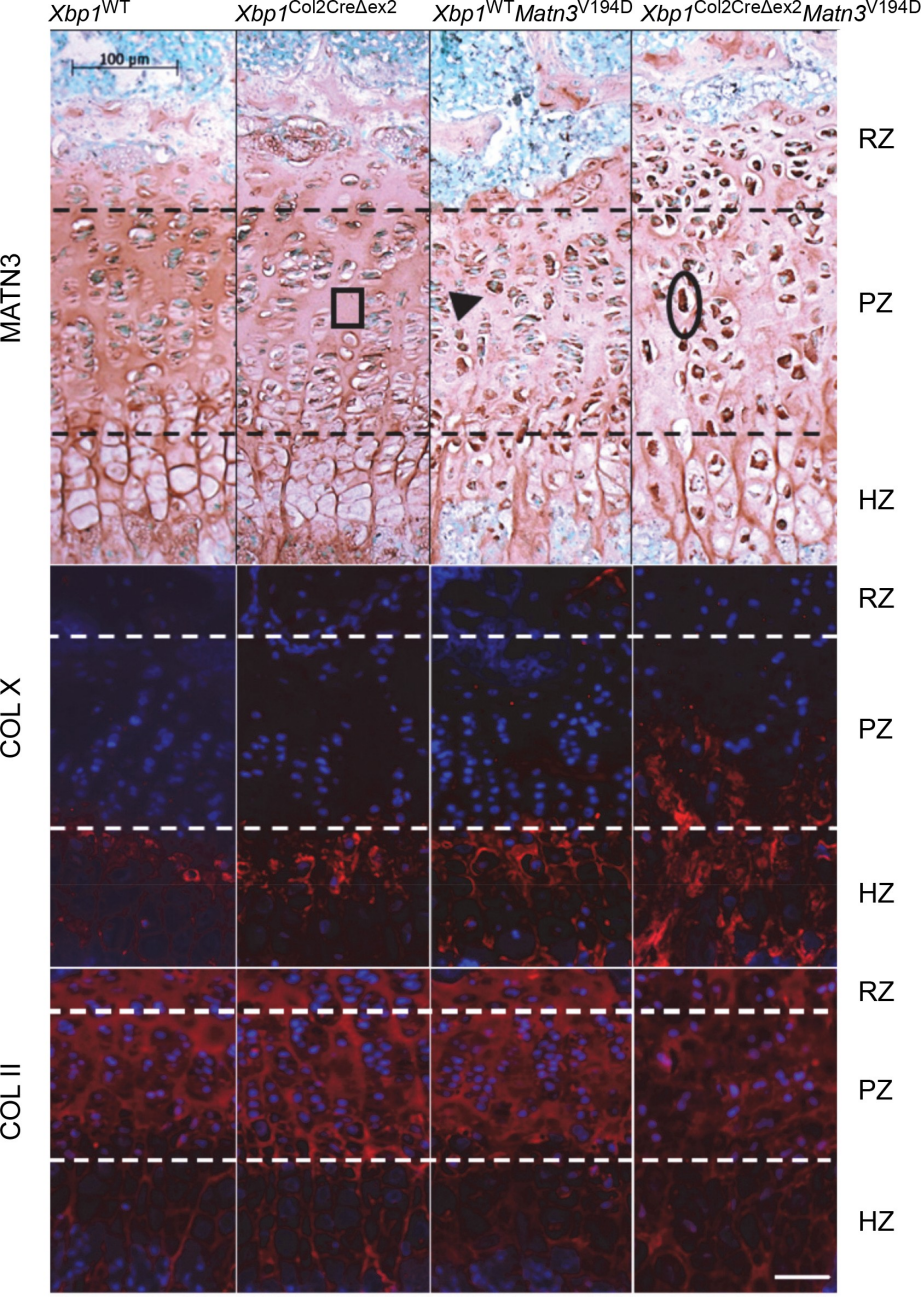
Immunohistochemical analysis of the cartilage growth plates at 3 weeks showed no difference in matrilin-3 (brown staining) amount or the typical extracellular distribution in the *Xbp1*^Col2CreΔex2^ tissue. The *Xbp1* null growth plates showed small areas of hypocellularity consistent with the previously reported decrease in proliferation (box). Mutant matrilin-3 was intracellularly retained (arrowhead) and depleted from the extracellular matrix in *Xbp1*^WT^ *Matn3*^V194D^ cartilage. Moreover, the columnar organisation of the proliferative one was affected and the cells adopted a more rounded morphology. The removal of XBP1 from EDM5 cartilage (*Xbp1*^Col2CreΔex2^ *Matn3*^V194D^) resulted in a dramatically aggravated phenotype with increased intracellular retention of matrilin-3 and the appearance of abnormally enlarged cells throughout the tissue (oval). Nuclear fast green was used as a counterstain. The zone of type X collagen (red) staining was broader in the *Xbp1* ^Col2CreΔex2^*Matn3*^V194D^ growth plates at 3 weeks, reflecting the growth plate disorganisation. Type II collagen was not affected in any of the analysed mouse models. DAPI was used as nuclear counterstain (blue).Key: RZ–resting zone, PZ–proliferative zone, HZ–hypertrophic zone, scale bar 100μm.

Deletion of XBP1 in the *Xbp1*^WT^ *Matn3*^V194D^ mouse line severely affected the morphology of the cartilage growth plates (Fig 2). Briefly, the *Xbp1*^WT^ and *Xbp1*^Col2CreΔex2^ growth plates presented with a typical and well-organised columnar arrangement of chondrocytes in the proliferative zone and an ordered progression from the resting to proliferative to hypertrophic cells along the vertical axis of the growth plate. The growth plates from *Xbp1*^Col2CreΔex2^ mice had a slightly reduced hypertrophic zone and small areas of hypocellularity, consistent with the previously published study [21]. In contrast, growth plates from *Matn3*^V194D^ mice were characterised by enlarged cells in the resting and proliferative zones due to retention of misfolded mutant matrilin-3 as previously described [33]. Finally, the growth plates from *Xbp1*^Col2CreΔex2^ *Matn3*^V194D^ mice had a dramatically altered morphology with abnormally enlarged cells present throughout the entire growth plate and concurrent with an apparent increase in the retention of mutant matrilin-3. This increased retention of mutant matrilin-3 appeared to correlate with an increase in amyloid-like intracellular deposits detected by Congo Red fluorescence (S2 Fig). The severe disorganisation of the growth plates from the *Xbp1*^Col2CreΔex2^ *Matn3*^V194D^ mice rendered impractical any measurement of the respective zones using histology images. However, the distribution of type X collagen (a marker of chondrocyte hypertrophy) in the *Xbp1*^Col2CreΔex2^ *Matn3*^V194D^ growth plates at 3 weeks was altered and collagen X staining extended into the proliferative zone, coinciding with the abnormally enlarged chondrocytes and indicating accelerated differentiation. In contrast, staining for type II collagen (a major component of the cartilage ECM) was unaffected (Fig 2).

### Chondrocyte apoptosis and proliferation are affected by the deletion of XBP1 in *Matn3*^V194D^ mice

**Fig 3:**
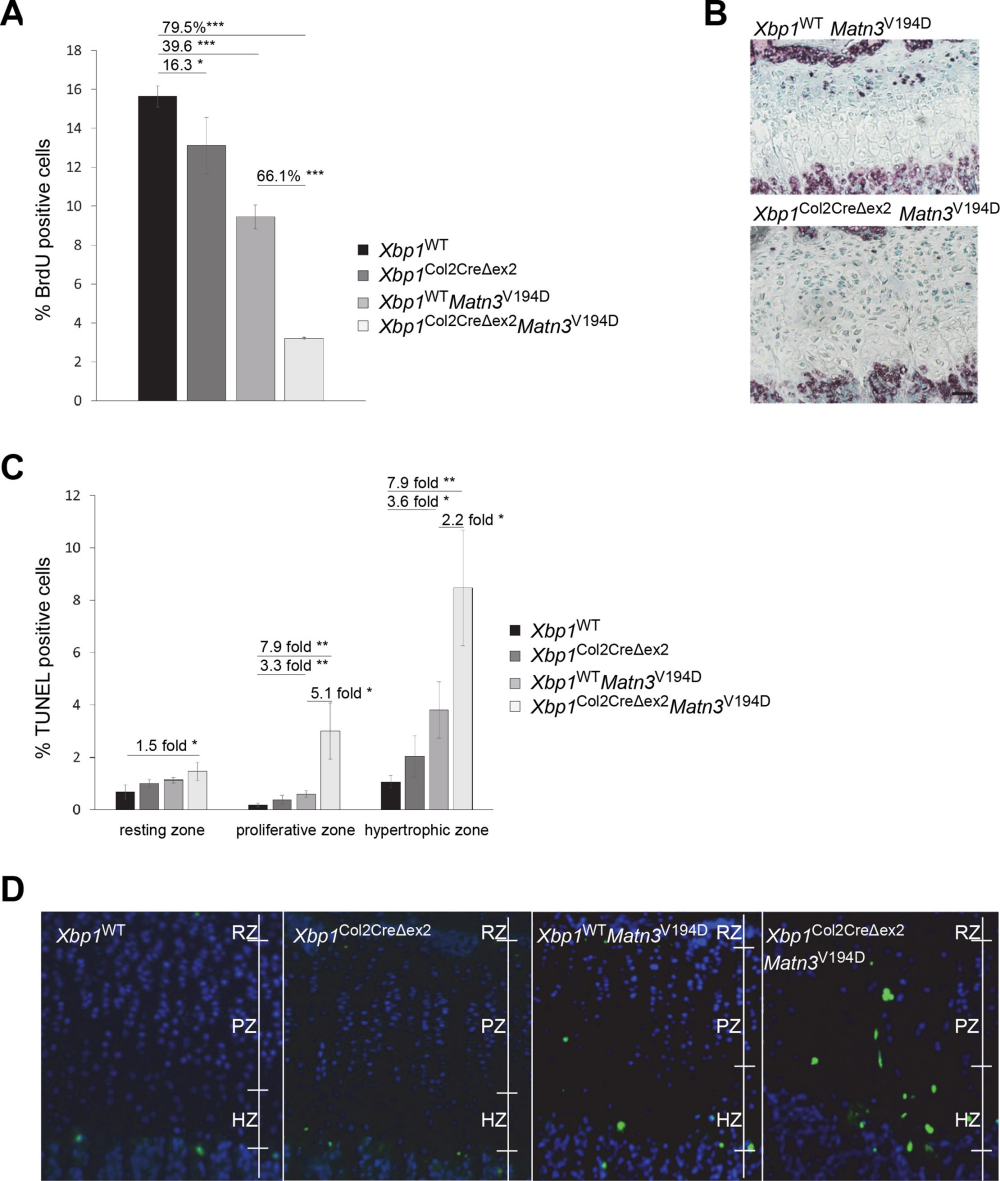
(A) 2h BrdU labelling pulse was performed to assess cell proliferation in the growth plate cartilage at 3 weeks of age. *Xbp1* null cartilage showed a 16% decrease in proliferation compared to the wild type control. The proliferation in the *Xbp1*^WT^ *Matn3*^V194D^ cartilage was decreased by nearly 40%. Proliferation levels in the *Xbp1*^Col2CreΔex2^ *Matn3*^V194D^ cartilage reflected the synergistic effect of the two genetic insults and was decreased by 66% (n = 3, One Way ANOVA). (B) The incorporation of the labelling agent (purple staining) into the proliferating cells was also decreased in the *Xbp1*^Col2CreΔex2^ *Matn3*^V194D^ chondrocytes indicating a slowed down cell cycle. Nuclear Fast Green was used as counterstain. (C) TUNEL assay was performed to assess apoptosis in cartilage growth plates at 3 weeks (n = 3, One Way ANOVA). Apoptosis was dramatically increased in all zones of the *Xbp1*^Col2CreΔex2^ *Matn3*^V194D^ growth plate. (D) The apoptosis, normally occurring at the lower hypertrophic zone in the cartilage growth plate, was also dysregulated in the *Xbp1*^Col2CreΔex2^ *Matn3*^V194D^ with cells dying in the proliferative and resting zones as well. Interestingly, the cells undergoing apoptosis correlated with the abnormally enlarged cellular morphology. Positive cells shown in green, DAPI used as nuclear counterstain (blue). Key: RZ–resting zone, PZ–proliferative zone, HZ–hypertrophic zone, * P<0.05, ** P<0.01, *** P<0.001, scale bar 100μm.

Chondrocyte proliferation in the growth plates of both *Xbp1*^WT^ *Matn3*^V194D^ and *Xbp1*^Col2CreΔex2^ mice was significantly reduced when compared to wild type controls as previously reported (~16% and ~40% decrease respectively) [21, 33]. By comparison, chondrocyte proliferation in *Xbp1*^Col2CreΔex2^ *Matn3*^V194D^ mice was decreased by ~80% when compared to the wild type mice and by ~66% when compared to *Xbp1*^WT^ *Matn3*^V194D^ mice suggesting a synergistic effect of the two mutations (Fig 3A). Moreover, the staining for BrdU not only showed a decrease in the relative number of BrdU positive cells, but also a reduced intensity of staining of these positive cells, suggesting a slower rate of DNA synthesis and an impaired cell cycle (Fig 3B).

Chondrocyte apoptosis in the growth plate was not affected by the deletion of XBP1 from cartilage as previously reported (*Xbp1*^Col2CreΔex2^ compared to *Xbp1*^WT^ controls)[21]. In contrast, the p.V194D *Matn3* mutation had a slight negative effect on the levels of apoptosis in the hypertrophic and proliferative zones, which is consistent with previous observations [33]. However, in the *Xbp1*^Col2CreΔex2^ *Matn3*^V194D^ double mutant mice there was a dramatic increase in the relative levels of chondrocyte apoptosis in the resting, proliferative and hypertrophic zones (1.5, ~8 and ~8-fold respectively compared to the wild type controls; Fig 3C). Indeed, TUNEL positive cells were found throughout the growth plates of *Xbp1*^Col2CreΔex2^ *Matn3*^V194D^ mice and appeared to correlate with the enlarged cell morphology previously noted in the histological analysis (Fig 3D).

### Comparative microarray analysis confirms that the XBP1 branch of the UPR has a role in modulating the aggregation of mutant matrilin-3 in the chondrocytes of *Matn3*^V194D^ mice

**Fig 4:**
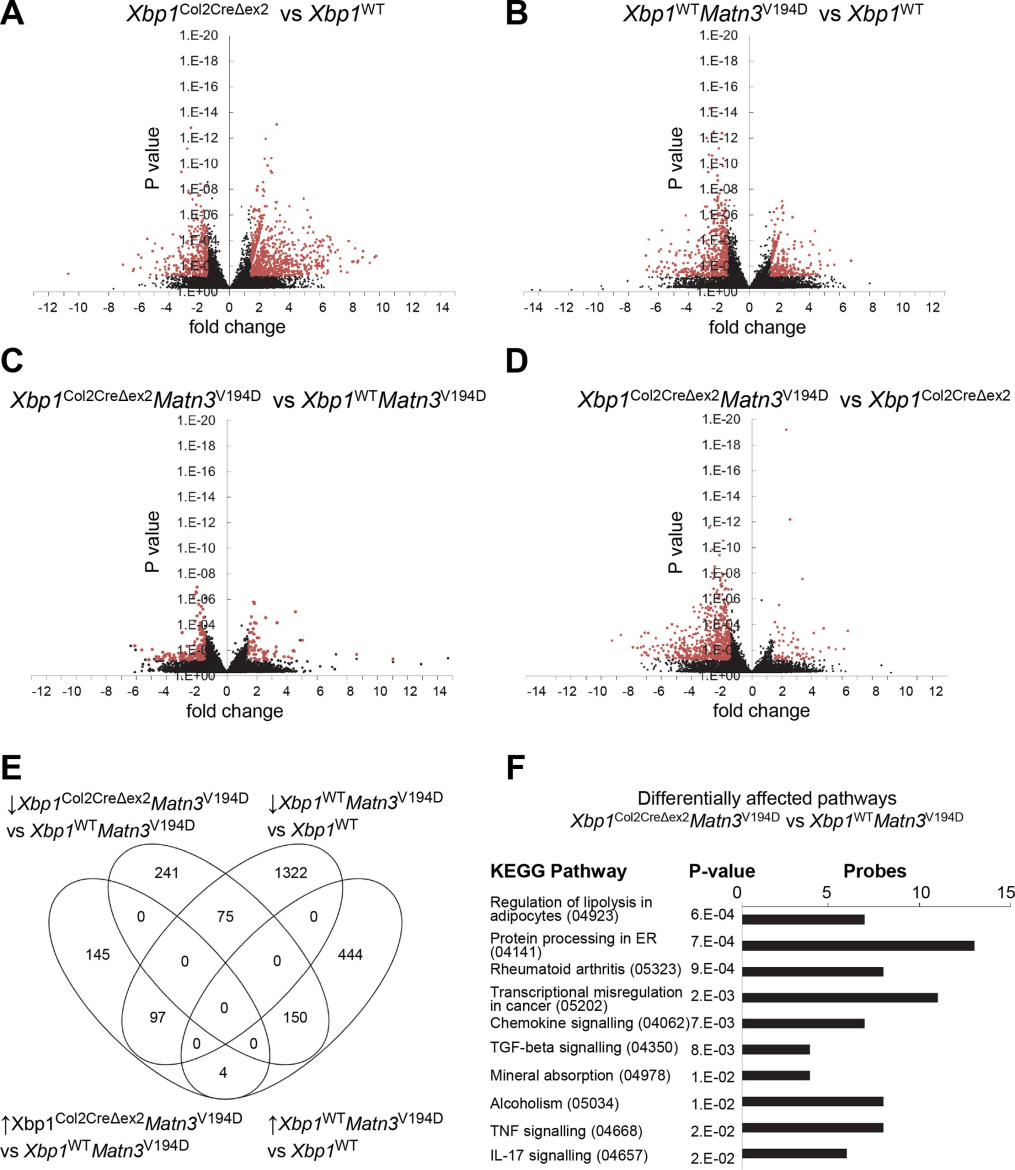
(A-D) Volcano plots of differential gene expression (in black) highlighting the significantly changed genes (1.5 fold, P<0.05; in red). (E) A Venn diagram comparing the significantly differentially expressed genes in the original EDM5 (*Xbp1*^WT^ *Matn3*^V194D^ vs *Xbp1*^WT^ *Matn3*^WT^) model and the effect of XBP1 deletion (*Xbp1*^Col2CreΔex2^ *Matn3*^V194D^ vs *Xbp1*^WT^ *Matn3*^V194D^). (F) Pathway analysis of the *Xbp1*^Col2CreΔex2^ *Matn3*^V194D^ vs *Xbp1*^WT^ *Matn3*^V194D^ microarray data was performed using the iPathwayGuide platform (Advaita Corp). Differentially expressed KEGG pathways are plotted using the P-values and the number of differentially expressed probes.

Deleting XBP1 from the chondrocytes of *Xbp1*^WT^ *Matn3*^V194D^ cartilage further exacerbated the disease phenotype, indicating that the XBP1 branch of the UPR pathway had a ‘chondroprotective’ role in proliferating chondrocytes. To further define the putative protective role of XBP1 we performed microarray analyses on mRNA derived from wild type and mutant animals with a comparable C57BL6 genetic background. Volcano plots showing the differential expression of genes and significant changes are shown in Fig 4A–4D and a heat map comparison is shown in S3 Fig.

In total 2092 genes were differentially expressed in mutant *Matn3* chondrocytes (*Xbp1*^WT^ *Matn3*^V194D^ vs *Xbp1*^WT^ analysis) compared to 2396 in XBP1 null cartilage (*Xbp1*^Col2CreΔex2^ vs *Xbp1*^WT^), 1316 in *Xbp1*^Col2CreΔex2^ *Matn3*^V194D^ vs *Xbp1*^Col2CreΔex2^ and finally 712 in *Xbp1*^Col2CreΔex2^ *Matn3*^V194D^ vs *Xbp1*^WT^ *Matn3*^V194D^ (S3 Fig). Overall, this indicated that both the presence of the matrilin-3 mutation and the absence of XBP1 were strong effectors of cartilage homeostasis.

The highest number of differentially expressed genes in common (1192) was noted for the cartilage-specific deletion of XBP1 (*Xbp1*^Col2CreΔex2^ vs *Xbp1*^WT^) and the p.V194D *Matn3* mutant (*Xbp1*^WT^ *Matn3*^V194D^ vs *Xbp1*^WT^) comparisons, suggesting that genes downstream of the XBP1 signalling pathway are key disease modulators in matrilin-3 related MED.

*Xbp1*^WT^ *Matn3*^V194D^ vs *Xbp1*^WT^ analysis confirmed the findings previously reported for this mouse line [5]. The 1494 genes downregulated in the mutant *Matn3* chondrocytes (*Xbp1*^WT^ *Matn3*^V194D^ vs *Xbp1*^WT^) were predominantly associated with regulation of gene expression, cell proliferation and apoptosis. A distinct subset of downregulated genes was associated with modulating the aggregation of mutant/misfolded proteins (*Cryab*, *Hspa1l*, *Hspb8*, *DNaja1*). Genes associated with cartilage and bone development (such as *Alpl*, *Col1a1*, *Col10a1*, *Evc*, *Hif1a*, *Igf1*, *Igfbp5*, *Ibsp*, *Mmp14*, *Nog*, *Pthlh*, *Smo* and *Tnc*) were also decreased. In contrast, 598 genes significantly upregulated in the *Matn3* mutant chondrocytes (*Xbp1*^WT^ *Matn3*^V194D^ vs *Xbp1*^WT^) were predominately associated with cell proliferation, migration and the cellular response to ER stress [5] and included genes such as *Creld2*, *Canx*, *Dnajc3*, *Grp94*, *Hyou1*, *Manf* (*Armet*), *Pdia3*, *Pdia4*, *Trib3* and *Xbp1* (S1 Table).

A *Xbp1*^Col2CreΔex2^ vs *Xbp1*^WT^ comparison confirmed the previously published involvement of Xbp1 in bone formation [21] and modulation of the ER stress response [34]. 1114 genes downregulated in XBP1 null cartilage (*Xbp1*^Col2CreΔex2^ vs *Xbp1*^WT^) were associated with transcription regulation, apoptosis, protein ubiquitination and cell cycle regulation [21]. Several genes involved in protein folding were also decreased including *Cryab*, *Dnajc4*, *Dnajc13*, *Dnajc22*, *Hspa4l* and *Hspa8*. 1282 genes upregulated in *Xbp1* null cartilage pertained to regulation of cell migration, angiogenesis and extracellular matrix organisation as previously published [21]. Genes for several ECM proteins including *Col1a1*, *Col10a1 Col4a1*, *Col4a2*, *Col18a1*, *Fbln5* and *Ibsp* were also upregulated, together with several signalling molecules such as *Fgf18*, *Fgf2*, *Notch1*, *Tgfb2* and *Vegfc*, indicating a deregulation of chondrocyte differentiation in the *Xbp1*^Col2CreΔex2^ mice.

A comparative analysis of genes differentially regulated by the *Matn3* mutation (*Xbp1*^WT^ *Matn3*^V194D^ vs *Xbp1*^WT^) with the genes differentially regulated by *Xbp1* ablation in *Matn3* mutant chondrocytes (*Xbp1*^Col2CreΔex2^ *Matn3*^V194D^ vs *Xbp1*^WT^ *Matn3*^V194D^) was undertaken to explain the dramatically exacerbated disease phenotype in the *Xbp1*^Col2CreΔex2^ *Matn3*^V194D^ mice and to identify *Xbp1*-dependent pathways in EDM5 (Fig 4E and 4F). Interestingly, the differentially changed genes are involved in cartilage differentiation/dedifferentiation pathways (“lipolysis in adipocytes”, “mineral deposition”, “TGFß signalling”, “rheumatoid arthritis”) and in the UPR (“protein processing in the endoplasmic reticulum”; Fig 4F). A detailed analysis revealed 57 genes decreased in the *Matn3* mutant chondrocytes and further decreased upon XBP1 deletion, indicating their expression is modulated downstream of XBP1. The GO terms for these genes included “protein folding”, “phospholipid biosynthesis process” and “nucleosome assembly” (S2 Table). The most highly and significantly represented GO term was “protein folding” and included the chaperone molecule DNAJA4, heat shock protein HSPA8 and crystallin alpha B (CRYAB) (S2 Table); all of which lie downstream of XBP1 signalling [34]. Moreover, the genes associated with “phospholipid biosynthesis process” included choline kinase alpha (*Chka*), ethanolaminephosphotransferase 1 (*Ept1*) and phosphatidylserine decarboxylase pseudogene 3 (*Pisd-ps3*), involved in the maintenance of vesicular membranes and in protein folding respectively.

150 genes were upregulated in the *Matn3* mutant cartilage (*Xbp1*^WT^ *Matn3*^V194D^ vs *Xbp1*^WT^) and downregulated upon removal of XBP1 (*Xbp1*^Col2CreΔex2^ *Matn3*^V194D^ vs *Xbp1*^WT^ *Matn3*^V194D^) indicating an ER-stress triggered XBP1*-*dependent response (S3 Table). The top GO terms associated with these were “response to toxic substance”, “response to lipopolysaccaride”, “regulation of ERK1 and ERK2 cascade” and “cell migration”. The genes changed in the “ERK1 and ERK2 signalling pathway” and “cell migration” (*Abl2*, *Cd44*, *Ccl5*, *Prkca*, *Sema7a*) are known to respond to extracellular signals and may be reflecting extracellular changes resulting from the intracellular stress. *Ltbp1*, one of the major players in the TGFß signalling, was dramatically increased in *Matn3* mutant cartilage (*Xbp1*^WT^ *Matn3*^V194D^ vs *Xbp1*^WT^) and decreased upon *Xbp1* removal, but unchanged in the XBP1 null control, indicating a stress-related response. Other interesting genes upregulated in *Matn3* mutant cartilage but downregulated upon removal of *Xbp1* were *Mmp3*, *Mmp10*, *Adam19* and *Sost*, suggesting potential changes in chondrocyte maturation and differentiation. Moreover, several genes upregulated in mutant *Matn3* cartilage and decreased upon removal of *Xbp1* (*Abcb1a*, *Creld2*, Pdia6, *Dnajc3*, *Ero1lb*, *Hao1*, *Hyou1*, *Magt1*, *Pex11a*, *Sdf2l1*, *Ugt1a1*) reflected changes in protein folding, disulphide bond formation, peroxisome activity and drug metabolism machinery implying an *Xbp1*-dependent non-canonical stress pathway is activated by the accumulation of misfolded aggregated matrilin-3. Interestingly, some of these genes were also implicated in the pathobiology of other aggregation-related diseases such as Alzheimer’s [17, 35].

A total of 79 genes were downregulated in *Matn3* mutant chondrocytes (*Xbp1*^WT^ *Matn3*^V194D^ vs *Xbp1*^WT^ analysis) but upregulated in *Matn3* mutant chondrocytes lacking XBP1 (*Xbp1*^Col2CreΔex2^ *Matn3*^V194D^ vs *Xbp1*^WT^ *Matn3*^V194D^) and the main GO term in this comparison was “osteoblast differentiation” (S4 Table). The genes associated with this grouping (*Col1a1*, *Igf1*, *Igfbp5*, *Ibsp*, *Tnc*) were downregulated by the presence of matrilin-3 mutation on both *Xbp1* backgrounds (wild type and null), indicating their downregulation in response to aggregation stress, and upregulated by the absence of XBP1 on wild type or matrilin-3 mutant background thereby suggesting XBP1 regulation [21].

Only 4 genes were increased in both *Xbp1*^WT^ *Matn3*^V194D^ vs *Xbp1*^WT^ and in *Xbp1*^Col2CreΔex2^ *Matn3*^V194D^ vs *Xbp1*^WT^ *Matn3*^V194D^ and these were *Serpina3c*, *Serpina3n*, *Ptger* and a hypothetical protein (2310033P09Rik).

### Differential expression profiling helps separate the aggregation-dependent and the XBP1-dependent molecular events

**Fig 5:**
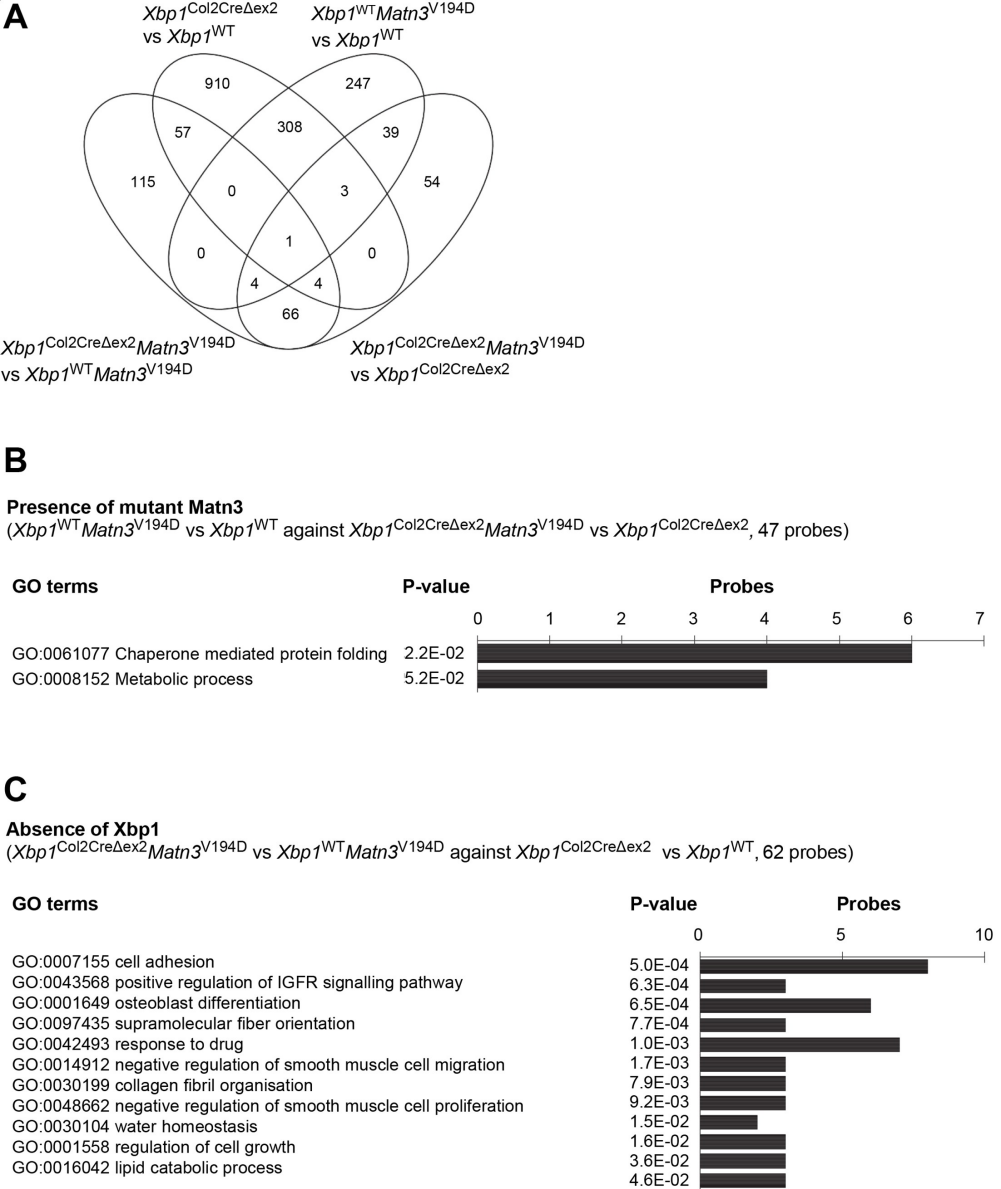
(A) Venn diagram showing an overlap in the significantly upregulated microarray probes from the 4 analyses (*Xbp1*^WT^ *Matn3*^V194D^ vs *Xbp1*^WT^, *Xbp1*^Col2CreΔex2^ *Matn3*^V194D^ vs *Xbp1*^Col2CreΔex2^, *Xbp1*^Col2CreΔex2^ *Matn3*^V194D^ vs *Xbp1*^WT^ *Matn3*^V194D^ and *Xbp1*^Col2CreΔex2^ vs *Xbp1*^WT^. (B) A graph showing the GO terms associated with 47 upregulated probes representing the XBP1 independent effect of a MATN3 mutation. (C) A graph showing the GO terms associated with 62 upregulated probes representing the MATN3 mutation independent effect of XBP1 deletion.

**Fig 6:**
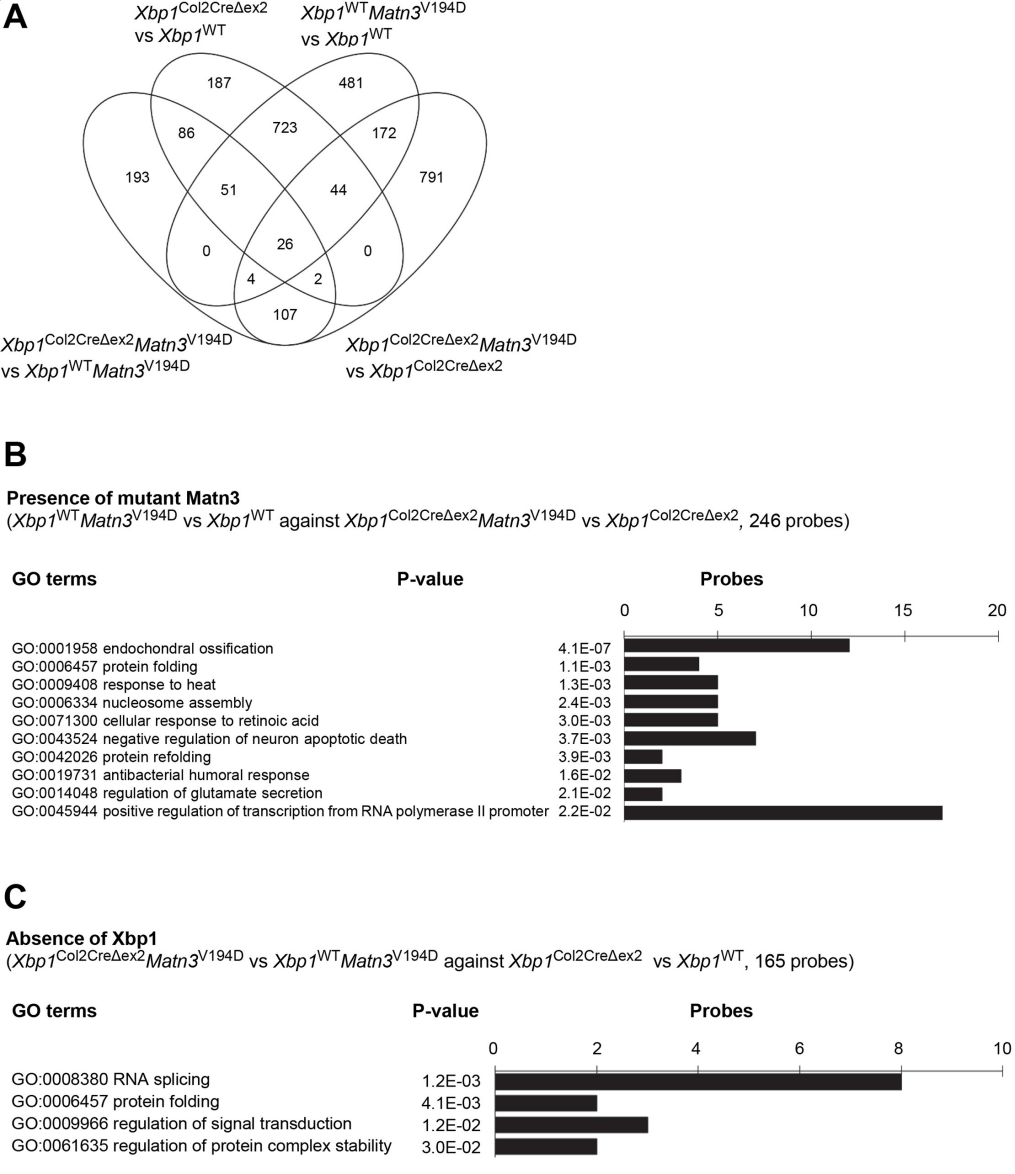
(A) Venn diagram showing an overlap in the significantly downregulated microarray probes from the 4 analyses (*Xbp1*^WT^ *Matn3*^V194D^ vs *Xbp1*^WT^, *Xbp1*^Col2CreΔex2^ *Matn3*^V194D^ vs *Xbp1*^Col2CreΔex2^, *Xbp1*^Col2CreΔex2^ *Matn3*^V194D^ vs *Xbp1*^WT^ *Matn3*^V194D^ and *Xbp1*^Col2CreΔex2^ vs *Xbp1*^WT^. (B) A graph showing the GO terms associated with 47 downregulated probes representing the XBP1 independent effect of a MATN3 mutation. (C) A graph showing the GO terms associated with 62 downregulated probes representing the MATN3 mutation independent effect of XBP1 deletion.

Expression profiling of the *Matn3* mutant cartilage (*Xbp1*^WT^ *Matn3*^V194D^ vs *Xbp1*^WT^) compared to the *Matn3* mutant cartilage on the XBP1-null background (*Xbp1*^Col2CreΔex2^ *Matn3*^V194D^ vs *Xbp1*^Col2CreΔex2^) was performed in order to elucidate the molecular events specifically dependent upon matrilin-3 mutation and independent of the UPR signalling downstream of XBP1 (Figs 5 and 6). 47 genes were upregulated (Fig 5B) and 246 genes downregulated (Fig 6B) in the presence of the matrilin-3 mutation. The upregulated genes clustered in “metabolic process” and “protein folding” GO terms and included *Bcat2*, *Lpcat1*, *Pnpla3* and *Uap1*, and *Atf5*, *Canx*, *Derl3*, *Manf*, *Pdia3*, *Pdia4* and *Pdia6* respectively. The downregulated genes pertained to “endochondral ossification” and included *Alpl*, *Col1a1*, *Col10a1*, *Cyr61*, *Dlx5*, *Gabbr1*, *Igf1*, *Ibsp*, *Mef2c*, *Mmp14*, *Pthlh* and *Tnc*; and “protein folding” including *Cryab*, *Dnaja1*, *Dnaja4*, *Hspa4l*, *Hspa8*, *Hsp90aa1*, *Tubb5*. Interestingly, a subset of ER-stress related genes downregulated independently of XBP1 in the presence of mutant matrilin-3 (*Cryab*, *Dnaja4*, *Hspa8* and *Hsp90aa1*) were further decreased in the *Xbp1*^Col2CreΔex2^ *Matn3*^V194D^ vs *Xbp1*^WT^ *Matn3*^V194D^ analysis and decreased in the *Xbp1* null cartilage (*Xbp1*^Col2CreΔex2^ vs *Xbp1*^WT^ analysis), indicating a synergistic effect of the two stressors and potential XBP1 modulation.

62 genes were upregulated (Fig 5C) and 165 genes downregulated (Fig 6C) following the removal of *Xbp1* from chondrocytes with or without the *Matn3* mutation (i.e. *Xbp1*^Col2CreΔex2^ vs *Xbp1*^WT^ compared to *Xbp1*^Col2CreΔex2^ *Matn3*^V194D^ vs *Xbp1*^WT^ *Matn3*^V194D^) indicating UPR-independent XBP1 signalling. The upregulated genes largely pertained to extracellular matrix organisation and osteoblast differentiation (e.g. *Col1a1*, *Igf1*, *Igfbp3*, *Igfbp5*, *Ibsp*, *Vegfc*), which is in agreement with previously published data [21], whereas the downregulated genes were clustered to RNA splicing (e.g. *Prpf38b*, *Rbm25*, *Rbm5*, *Cpsf6*, *Hspa8*, *Mbnl1*, *Srrm2*, *Tra2a*) and protein folding (*Ahsa1*, *Pdia6*).

### Aggregation-specific ER-resident chaperones are differentially expressed in chondrocytes from the MCDS and EDM5 mice, delineating a differentiation state specific stress response

Removal of *Xbp1* had a profound effect on the phenotype of EDM5 mice, which is a disease predominantly affecting chondrocytes undergoing proliferation, but in contrast, deletion of *Xbp1* had no effect on the severity of MCDS, a disease of the hypertrophic zone of the growth plate [31]. We therefore assessed the modulation of the UPR machinery in different zones by comparing entire growth plate microarrays (*Xbp1*^*Col2CreΔex2*^ vs *Xbp1*^WT^) against a dataset generated for the hypertrophic zone only (*Xbp1*^*Col2CreΔex2*(HZ)^ vs *Xbp1*^WT(HZ)^; GEO series accession number GSE72261).

Interestingly, both the hypertrophic zone alone and the full cartilage growth plate showed unique gene expression signatures upon removal of *Xbp1* (S3 Fig). For example, whilst *Pdia6* was decreased in both the hypertrophic zone and the full growth plate following XBP1 ablation, the genes encoding other aggregation-specific chaperones such as *Cryab*, *Dnaja4*, *Hspa1l* and *Hspa8* were decreased in chondrocytes from the whole growth plate, but not in hypertrophic zone chondrocytes alone, suggesting differentiation-state dependent XBP1 modulation. In contrast, ATF6 signalling appeared to be affected by the deletion of XBP1 in both the whole growth plate and the hypertrophic zone alone. In the hypertrophic zone this resulted in an upregulation of the more stable, but weaker activator ATF6ß, whereas the full growth plate analysis showed an increase in ATF6ɑ and no change in ATF6ß, potentially indicating a decrease in ATF6ß in the proliferative zone and a differentiation-state dependent modulation of ATF6 signalling.

We then compared the microarray data generated using hypertrophic chondrocytes from the MCDS mice [36] with the microarray data of chondrocytes from the EDM5 mice to determine the cellular response to the retention and aggregation of mutant protein (S3 Fig). 586 differentially expressed genes were shared between the MCDS (*Col10a1*^*N617K*^ vs. *Col10a1*^WT^) and EDM5 (*Matn3*^V194D^ vs. *Matn3*^WT^) cartilage. 136 genes were upregulated in both mice and the main GO term for these was “ER response” and included the following genes, *Atf5*, *Atf6*, *Creld2*, *Derl3*, *Dnajc3*, *Hsp90b1* (*Grp94*), *Hyou1*, *Manf*, *Pdia3*, *Pdia4*, *Trib3* and *Xbp1*. Of these genes *Creld2*, *Dnajc3* and *Hyou1* appeared to be controlled by XBP1 under ER stress as suggested by their downregulation in the *Xbp1*^*Col2Δex2*^ *Matn3*^V194D^ vs. *Xbp1*^WT^ *Matn3*^V194D^ comparison. In contrast, *Atf5*, *Derl3*, *Hsp90b1*, *Manf* and *Pdia4* were all upregulated or unchanged in *Xbp1*^*Col2Δex2*^ *Matn3*^V194D^ vs. *Xbp1*^*Col2Δex2*^ and in *Xbp1*^WT^ *Matn3*^V194D^ vs. *Xbp1*^WT^ comparisons, indicating an XBP1-independent effect of mutant protein misfolding/aggregation. Interestingly, several of these genes have previously been shown to be downstream of ATF6 signalling ([34]; Table 1). 169 genes were downregulated in both comparisons and the top GO term for these was “skeletal development”, including genes such as *Alpl*, *Bcan*, *Cebpd*, *Col1a1*, *Crlf3*, *Deaf1*, *Foxa2*, *Ibsp*, *Kazald1*, *Mmp14*, *Nog*, *Smo* and *Sox4*. Many of these represent a delay in terminal differentiation, which could explain the phenotype of short-limbed dwarfism in both mouse models. ER stress related genes that were differentially expressed between the two models included *Ahsa1*, *Cryab*, *Hspa8*, *Hsp90aa1* and *Hsph1*, all of which were downregulated in *Matn3* mutant (*Matn3*^V194D^ vs. *Matn3*^WT^) cartilage and upregulated in the *Col10a1* mutant (*Col10a1*^*N617K*^ vs. *Col10a1*^WT^) comparison. These were downregulated in mice expressing mutant matrilin-3 in both the presence and absence of XBP1, but were further downregulated upon ablation of *Xbp1* indicating that they are ER-stress responsive and XBP1-dependent. Moreover, the transcriptomic comparison of EDM5 and MCDS growth plate chondrocytes suggested specific involvement of ATF6 and XBP1 signalling pathways in the UPR responses in the EDM5 mouse model, and an ATF6 and PERK-specific response in the MCDS cartilage (Table 1).

**Table 1:** A table summarising microarray data of the differential expression of representative IRE1ɑ (XBP1), ATF6 and PERK target genes [34, 37–40] in the EDM5 and MCDS models, and in EDM5 upon removal of XBP1 (EDM5/X). n.s. = not significant.

**Xbp1 target genes**	**EDM5**	**EDM5/X**	**MCDS**
*Abcb1a*	4.57	-3.19	n.s.
*Edem3*	n.s.	-2.28	2.64
*Hao1*	16.67	-2.60	n.s.
*Pdia6*	1.60	-1.86	4.93
*Pex11a*	1.62	-1.99	n.s.
*Ugt1a1*	1.90	-3.47	n.s.
**ATF6 target genes**	**EDM5**	**EDM5/X**	**MCDS**
*Canx*	1.86	n.s.	1.87
*Derl3*	25.20	n.s.	19.7
*Ero1l*	1.86	n.s.	8.57
**PERK target genes**	**EDM5**	**EDM5/X**	**MCDS**
*Ddit3*	-2.28	n.s.	24.25
*Fads3*	n.s.	n.s.	3.25
*Herpud1*	n.s.	n.s.	4.59
*Hspa9*	n.s.	n.s.	5.28
*Leprotl1*	n.s.	n.s.	3.48
*Steap1*	n.s.	n.s.	12.99
**Genes downstream of ATF6 and/or PERK**	**EDM5**	**EDM5/X**	**MCDS**
*Manf*	2.13	n.s.	3.03
*Pdia3*	2.28	n.s.	2
*Pdia4*	2.52	n.s.	16
**Genes downstream of Xbp1 and/or ATF6**	**EDM5**	**EDM5/X**	**MCDS**
*Creld2*	2.84	-2.32	7.46
*Dnajc3*	3.98	-1.52	3.73
*Grp94*	1.86	n.s.	9.85
*Hyou1*	3.74	-1.66	4.59
*Xbp1*	2.07	-1.64	2.82
**Genes downstream of synergy of ATF6 and Xbp1**	**EDM5**	**EDM5/X**	**MCDS**
*Derl1*	n.s.	n.s.	4.59
*Derl2*	n.s.	n.s.	1.51
*Edem1*	n.s.	n.s.	2.83
*Edem2*	n.s.	n.s.	n.s.
*Sel1l*	n.s.	n.s.	4.59
*Syvn1*	n.s.	n.s.	3.03
*Vcp*	n.s.	n.s.	1.51

**Fig 7:**
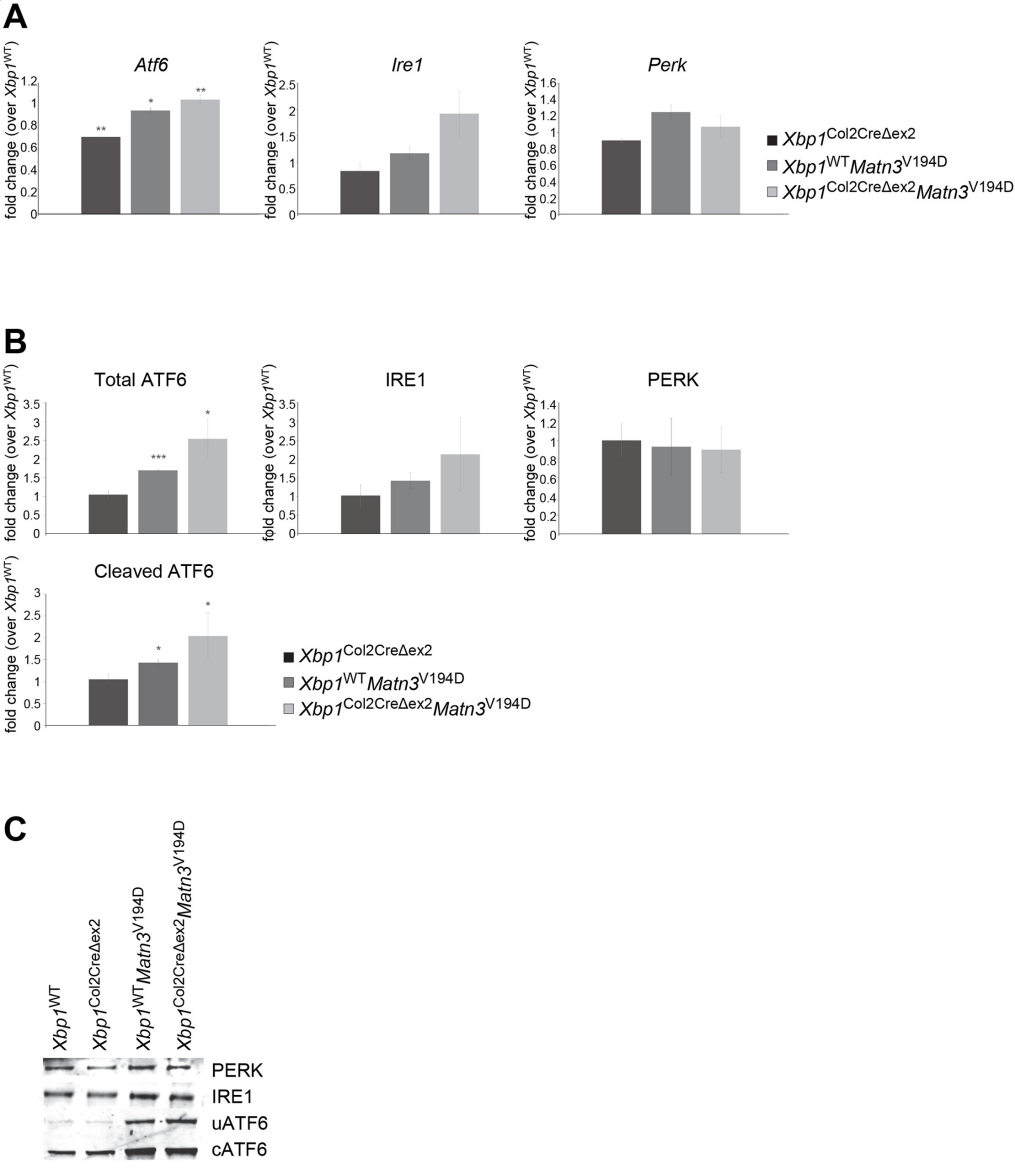
(A) Real time RT-qPCR data verifying expression levels of *Atf6*, *Ire1* and *Perk* using mRNA extracted from 5 day old chondrocytes (n = 3, Student t-test). No detectable changes in *Perk* expression confirm lack of involvement of the PERK signalling pathway in EDM5 pathobiology. (B) Densitometry measurement of 3 biological replicates of Western blotting for ATF6, IRE1 and PERK, showing no difference in IRE1 and PERK protein levels in EDM5 cartilage with or without XBP1. Interestingly, the amount of total and active (cleaved) ATF6 was dramatically increased in EDM5 cartilage compared to wild type controls and upon further XBP1 deletion. (C) Representative Western blotting images. Key: * P<0.05, ** P<0.01, *** P<0.001, scale bar 100μm.

In order to further confirm the lack of PERK involvement in EDM5 pathobiology, the UPR data obtained from the microarray analysis were verified by quantitative real-time RT-PCR analysis of *Ire1*, *Atf6*, *Perk* (Fig 7A). Interestingly, the expression levels of *Ire1* and *Perk* were unchanged in all genotypes analysed, when compared to the wild type controls. Western blotting of total cartilage homogenates was used to verify the protein levels of IRE1, ATF6 and PERK (Fig 7B and 7C). Individual blots and loading controls are shown in S4 Fig. The results of densitometry analysis of the Western blots corresponded to the qPCR data and showed no difference in PERK and IRE1 protein levels across the genotypes, and an increase in ATF6 protein levels in EDM5 cartilage (1.7 fold), with a corresponding 1.4 fold increase in the amount of the active (cleaved) ATF6, further confirming the activation of the ATF6 signalling branch of the UPR in EDM5 tissue. ATF6 protein levels were also elevated in EDM5 cartilage lacking XBP1 (2.5 fold) when compared to wild type controls but were not statistically different when compared to EDM5 samples.

**Fig 8:**
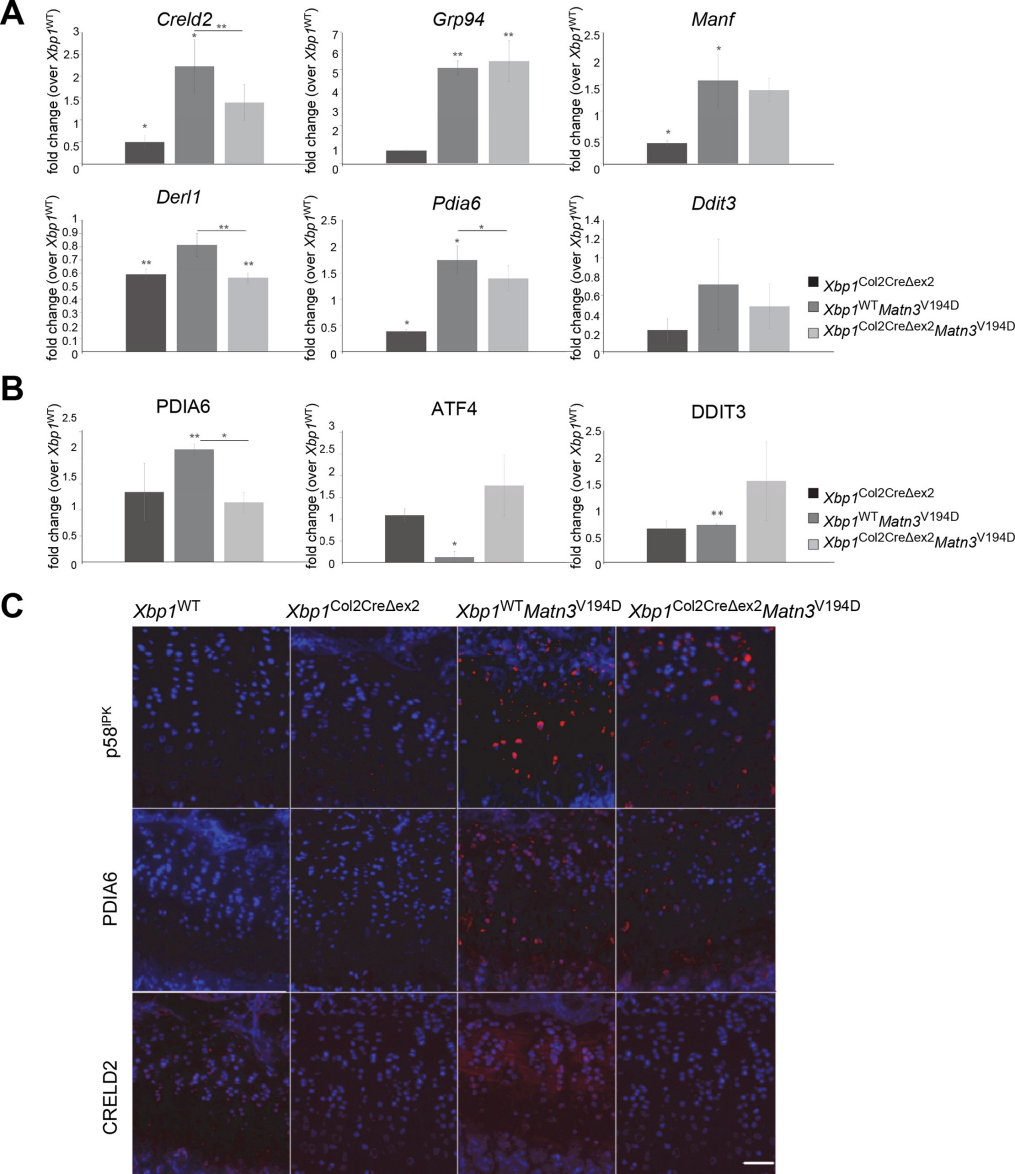
(A) Real time RT-qPCR of genes downstream of XBP1, XBP1/ATF6 and PERK signalling. *Creld2*, *Derl1* and *Pdia6* (downstream of XBP1) follow an XBP1 dependent pattern of expression, and are upregulated in EDM5 cartilage and decreased upon deletion of XBP1. *Grp94* and *Manf* (downstream of ATF6 and/or XBP1) appear to be ATF6-dependent in EDM5 cartilage and are not affected by lack of XBP1 in *Xbp1*^Col2CreΔex2^ *Matn3*^V194D^ tissue. The mRNA levels of *Ddit3* (*Chop*), a downstream effector of PERK, are not changed in any of the mouse lines analysed. (B) Densitometry measurement of 3 independent biological replicates of Western blotting for PDIA6 (downstream of XBP1), and ATF4 and DDIT3 (CHOP) (both downstream of PERK), showing a decrease in PERK effector levels in EDM5 cartilage. (C) Immunohistochemical staining of 3 week old cartilage growth plates showing a decrease in p58^IPK^, PDIA6 and CRELD2 staining (red) upon XBP1 deletion in the EDM5 cartilage. DAPI (blue) was used as a counterstain (blue). Key: * P<0.05, ** P<0.01, scale bar 100μm.

We also assessed the expression levels of selected downstream targets of the IRE1, ATF6 and PERK signalling branches of the UPR in mRNA isolated from primary chondrocytes extracted from cartilage (Fig 8A). These genes included *Pdia6* (downstream of *Xbp1*), *Creld2* (regulated by *Atf6* and *Xbp1*), *Derl1* (downstream of synergistic actions of *Xbp1* and *Atf6*), *Grp94* (downstream of *Atf6*), *Manf* (regulated by *Atf6* and *Perk*), and *Ddit3* (downstream of *Perk*). Interestingly, whilst the levels of *Xbp1* and *Atf6* effectors were increased (*Creld2* 2.2-fold, *Grp94* 5.1 fold, *Manf* 1.6 fold, *Pdia6* 1.7 fold), the levels of *Perk* and of *Ddit3*, a pro-apoptotic gene specifically regulated by PERK, were not changed in the EDM5 tissues, further confirming the specific ATF6 and XBP1 involvement in EDM5 pathobiology. In addition, genes that are regulated or co-regulated by *Xbp1* (*Creld2*, *Derl1*, *Pdia6*) were downregulated in EDM5 chondrocytes upon XBP1 removal, whereas genes downstream of *Atf6* (*Grp94*, *Manf*) and *Perk* (*Ddit3*) were unchanged, further confirming our hypothesis. Moreover, Western blotting of total cartilage homogenates was used to verify the protein levels of PDIA6 (downstream of IRE1/XBP1), and CHOP (DDIT3) and ATF4 (both downstream of PERK), in *Xbp1*^WT^, *Xbp1*^WT^ *Matn3*^V194D^, *Xbp1*^*Col2Δex2*^ and *Xbp1*^*Col2Δex2*^ *Matn3*^V194D^ cartilage (Fig 8B). The results of densitometry analysis of the Western blots were consistent with the expression levels seen in the mRNA analysis and showed a 2.3 fold increase in PDIA6 levels in EDM5 cartilage and a decrease to wild type levels upon XBP1 deletion. Moreover, the densitometry analysis further confirmed the lack of involvement of the PERK branch of the UPR in the EDM5 disease signature and showed suppression of PERK mediated signals (both ATF4 and CHOP protein levels were decreased in EDM5 cartilage, 0.8 and 0.4 fold, respectively). Changes in the expression levels of ATF6/XBP1 regulated chaperone CRELD2, aggregation-related chaperone protein PDIA6 and proliferation regulator p58^IPK^ (DNAJC3) were further verified in wild type and mutant cartilage growth plates by immunohistochemistry (Fig 8C). All three of these effectors were upregulated in the p.V194D *Matn3* mutant cartilage and decreased upon deletion of XBP1, indicating XBP1-dependence of EDM5 UPR signalling. Interestingly, p58IPK has been shown to dampen PERK signalling and attenuate eIF2ɑ phosphorylation [40, 41]. Moreover, it has been shown that PERK can modulate *Xbp1* expression and splicing in response to mutant protein aggregation [42, 43]. This effect of PERK signal on IRE1ɑ activity and splicing of *Xbp1* can be evidenced by a decreased ratio of alternatively spliced *Xbp1* (*Xbp1*^s^) to unspliced *Xbp1* (*Xbp1*^u^) in the EDM5 compared to the MCDS chondrocytes (S3 Fig).

## Discussion

The IRE1ɑ/XBP1 signalling branch is the most conserved branch of the UPR, essential in cellular response to the accumulation of misfolded proteins by regulating chaperone protein expression and ER-associated protein degradation (ERAD). Most likely due to the high protein secretory burden of chondrocytes and osteoblasts and the need for robust ER machinery during long bone growth [44, 45], this pathway is also important for skeletal development and was shown to regulate osteoblast differentiation *in vitro* [44] and chondrocyte proliferation and bone mineralisation *in vivo* [21]. XBP1 is the main effector of the pathway and *Xbp1* gene is non-conventionally spliced by autophosphorylated IRE1ɑ following its dissociation from the main UPR sensor BiP. The spliced form (XBP1s) then translocates to the nucleus to act as a transcription factor and activates genes encoding chaperones and ERAD components [7]. The unspliced form (XBP1u) has a shorter half-life and can shuttle between the nucleus and the cytoplasm where it can aid in proteasomal degradation of XBP1s protein and/or the active form of ATF6 thereby acting as a regulator of UPR signalling [46, 47].

Xbp1s mRNA has been identified in the ‘disease signature’ of many conditions resulting from the ER retention of misfolded mutant protein [34] and in particular several diseases characterised by the formation of insoluble intracellular aggregates such as type II diabetes [20], Alzheimer’s disease [17, 35], Huntington’s disease [18, 19], metaphyseal chondrodysplasia type Schmidt (MCDS) [6, 36] and matrilin-3 related multiple epiphyseal dysplasia (EDM5) [5, 33], suggesting XBP1-dependent modulation of ER stress in these conditions. More specifically, it has previously been shown that MCDS and EDM5 share a common disease signature consistent with a classical UPR and defined by an upregulation of ATF6, alternative splicing of *Xbp1* and an increase in *Canx*, *Creld2*, *Derl3*, *Dnajc3*, *Hyou1*, *Manf*, *Pdia3*, *Pdia4*, *Pdia6* and *Xbp1* gene expression [32]. It was therefore surprising that deletion of XBP1 in chondrocytes of a MCDS mouse model had no effect on the severity of the skeletal phenotype [31]. In contrast, deleting XBP1 from chondrocytes in the mouse model of EDM5 resulted in a dramatic increase in disease severity with significantly shorter limbs, deformed ribcages, severely disrupted cartilage growth plates and increased retention of mutant matrilin-3, suggesting an important role for XBP1 in response to abnormal protein aggregation in proliferating chondrocytes. Chaperone proteins involved in the processing of insoluble intracellular aggregates and already decreased in EDM5 chondrocytes (such as CRYAB, DNAJA1, DNAJA4, HSP1L and HSPA8), were further decreased following the deletion of XBP1. Interestingly, several of these were differentially expressed between the MCDS and EDM5 mice, indicating that the differentiation state of certain cells can influence their response to aggregation of mutant misfolded protein [36]. Moreover, several genes pertaining to proteasomal degradation (*Derl1*, *Derl2*, *Edem1*, *Edem3*) and autophagy (*Atg2b*, *Atg4b*, *Atg5*, *Atg10*, *Atg12*, *Atg13*) were upregulated in MCDS chondrocytes, but not EDM5 chondrocytes, indicating that despite toxic protein aggregation MCDS chondrocytes were able to upregulate crucial components of the degradation machinery. Interestingly, a recent study showed that the proteasomal and autophagy inducer carbamazepine can enhance these responses and reduce the intracellular retention of mutant collagen X and restore long bone growth in MCDS mice [48].

**Fig 9:**
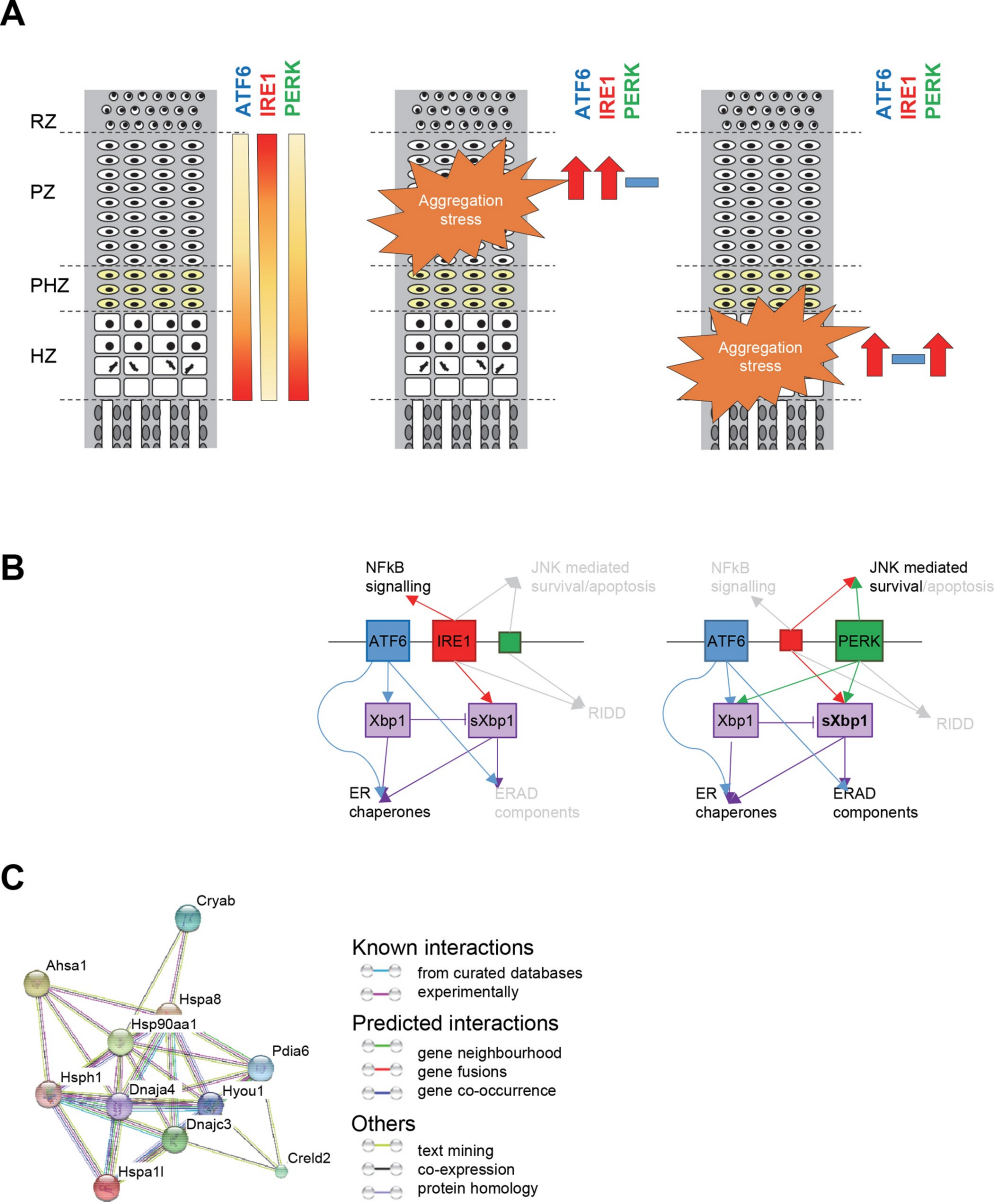
(A) Schematic explaining the basal levels of expression of the UPR sensors in the different zones of the cartilage growth plate and their differential expression upon the protein aggregation insult. (B) A schematic presenting the cross-talk of the UPR signalling branches upon misfolded protein aggregation in the proliferative (EDM5) and hypertrophic (MCDS) zones. (C) STRING visualisation of XBP1 modulated genes differentially expressed in the 5 day old EDM5 cartilage compared to the wild type control showing the postulated “aggregosome”.

The differences in the response to prolonged/chronic ER stress between proliferative and hypertrophic chondrocytes might stem from differentiation-specific variances in the basal levels of the three canonical ER stress pathways in the different zones of the cartilage growth plate. In physiological conditions the levels of ATF6ɑ and PERK are increased in hypertrophic chondrocytes compared to proliferative chondrocytes, whilst IRE1ɑ signalling appears more important in the proliferative zone of the growth plate (Fig 9A, [36, 49, 50]). It has been shown that the deletion of the ɑ or the ß isoform of ATF6 differentially affects the cartilage growth plate both in physiological conditions and following disease-associated ER stress, further confirming a modulation of the UPR through the differentiation state of a chondrocyte [51]. Interestingly, the levels of XBP1 are also increased in chondrocytes of the normal hypertrophic zone, potentially due to upregulated ATF6 signalling associated with hypertrophy [52, 53]. It is therefore not surprising that upon the induction of ER stress, ATF6ɑ and PERK were further upregulated in the hypertrophic MCDS chondrocytes. *Xbp1* expression was induced in the hypertrophic cells of MCDS mice and *Xbp1* was alternatively spliced, although the levels of IRE1ɑ did not increase (Fig 9A and 9B, [36]). Expression of genes downstream of ATF6 (including *Xbp1* [54]) and PERK (*Ddit3*, *Cebpb*), as well as the expression of ERAD genes requiring the synergy of XBP1s and ATF6 signalling were upregulated in *Col10a1*^*N617K*^ mice (Table 1, [55]). However, this was not sufficient in itself to trigger the ERAD pathway as evidenced by the increased intracellular retention of type X collagen in MCDS chondrocytes and the observation that MCDS mice lacking *Xbp1* showed no increase in disease severity [31].

In contrast, ER/cell stress induced by the aggregation of misfolded mutant matrilin-3 protein in proliferative chondrocytes of EDM5 mice resulted in upregulation of *Atf6a*, and upregulation of protein levels of ATF6 and its downstream effectors (GRP94, MANF). Expression level of *Ern1* (gene encoding IRE1ɑ) was not changed in EDM5 cartilage; however, the IRE1ɑ activity was enhanced, as evidenced by increased Xbp1 splicing and upregulation of XBP1 effectors (CRELD2, PDIA6). Interestingly, the levels of PERK and its downstream targets (ATF4, DDIT3) were not increased in EDM5 cartilage. ATF6 and XBP1 can form heterodimers and several UPR effectors can be modulated by either XBP1 or ATF6 or only by the actions of both [37, 55]. Interestingly, the expression of *Dnajc3*, which is downstream of XBP1 and ATF6, was increased in EDM5 chondrocytes and potentially further dampened PERK signalling [40, 41], as evidenced by a decrease in protein levels of PERK downstream effectors, ATF4 and DDIT3 in EDM5 cartilage. We therefore hypothesise that the UPR response in chondrocytes of the proliferative zone is primarily due to interplay between ATF6 and IRE1ɑ signalling and is not influenced by PERK. Therefore, the interplay between the XBP1 and ATF6 signalling is crucial for the pathobiology of aggregation-related diseases and that the modulation of the XBP1 pathway may present a promising therapeutic target.

The potential role of PERK modulation of *Xbp1* expression and *Xbp1* splicing in response to mutant protein aggregation in hypertrophic cells is an interesting aspect that requires further investigation [42, 43]. The effect of PERK signalling on IRE1ɑ activity and splicing of *Xbp1* can be evidenced by a decreased ratio of *Xbp1*^s^:*Xbp1*^u^ in the EDM5 compared to the MCDS chondrocytes. It is therefore interesting to speculate that inducing the upregulation of PERK signalling in EDM5 chondrocytes may lead to a preferential upregulation of XBP1-dependent ERAD or autophagy [43]. The upregulation of many ERAD and autophagy components in the MCDS cartilage, and the ability of carbamazepine to induce degradation of mutant collagen X, may also be a result of the hypertrophic chondrocytes being “ERAD primed” through their differentiation process (Fig 9B, [36, 56, 57]).

Several of the XBP1-dependent genes identified in our EDM5 study play a role in an intracellular pathway for the removal of xenobiotics and lipophilic substances. In particular *Abcb1*, *Ugta1a1* and *Hao1* were upregulated in the EDM5 chondrocytes and downregulated upon removal of *Xbp1*, indicating an alternative XBP1-dependent pathway triggered by the aggregation of mutant protein. STRING visualisation of the UPR genes differentially expressed in EDM5 cartilage and modulated by XBP1 reveals a potential experimentally supported “aggregosome” (Fig 9C). Interestingly, this disease signature is similar to Alzheimer’s disease (AD) models with intracellular retention of amyloid-like deposits. Specifically, *Creld2*, *Dnajc3*, *Manf*, *Pdia3* and *Pdia6* are upregulated both in EDM5 and in AD, and *Cryab*, *Dnaja4* and *Hsph1* are downregulated in both conditions whilst ERAD components are not affected [58]. CRYAB and HSPH1 are both chaperone proteins that prevent aggregation of mutant proteins and defects in CRYAB have been associated with Huntington’s disease and Alzheimer’s disease, where its levels decrease in age-dependent manner [59–61]. It is therefore interesting to speculate that these two chaperones could represent potential therapeutic targets for EDM5 and other protein aggregation related diseases. In fact, overexpression of CRYAB in a mouse model of Huntington’s disease was shown to be neuroprotective and reduced the size of aggregate inclusions in the affected brains [62].

Finally, a polymorphism in the *Xbp1* promoter is one of the risk factors for Alzheimer’s disease [17] and the pathobiology of protein aggregation conditions such as Alzheimer’s and Huntington’s disease can be regulated via a modulation of the IRE1ɑ-XBP1 branch of the UPR [18, 19, 35]. Our data suggests that the pathobiology of EDM5 is governed by the tight regulation between the IRE1ɑ and ATF6 signalling and a balance between the spliced and unspliced forms of *Xbp1*. It is therefore interesting to speculate that modifying the XBP1 signalling pathway via chemical or genetic intervention might present a therapeutic avenue for a broader range of protein aggregation diseases, leading to an increase in ERAD or autophagy, or an increased sensitivity to degradation-inducing therapies such as carbamazepine treatment. Several studies in normal cells, cancer and disease models have previously identified chemicals that activate or block the RNAse and/or kinase activity of IRE1ɑ and modulate the *Xbp1*^s^:*Xbp1*^u^ ratio as potential therapeutic modifiers of the IRE1ɑ/XBP1 signalling pathway [63–66]. This study further confirms the importance of this pathway in degradation of insoluble intracellular aggregates and offers a novel therapeutic avenue that could be applicable to a broader range of aggregation conditions.

## Materials and methods

### Generation of mouse lines

*Xbp1*^WT^ *Matn3*^V194D^ mice [33] were crossed with *Xbp1*^*Col2CreΔex2*^ mice [21] in which Xbp1 mRNA is inactivated by the Col2a1 promoter-driven Cre recombinase-mediated deletion of exon 2 to generate the compound mutant mouse line, *Xbp1*^*Col2CreΔex2*^ *Matn3*^V194D^. All the mice were generated on the C57BL6/J background to control for the background effects. Genotyping was performed as previously described and RT-PCR and sequencing were performed on cartilage RNA to confirm deletion of *Xbp1* exon 2 in mutant chondrocytes.

### Ethics statement

All experiments were approved by the University of Manchester Animal Ethical Review Group and performed in compliance with the Scientific Procedures Act of 1986 and the relevant Home Office (under PPL 40/2884 and PPL60/04525) and Institutional regulations governing animal breeding and handling.

### Bone measurements

Mice of different genotypes (>5 per age per genotype) were sacrificed at 3, 6 and 9 weeks of age and X-rayed using Faxitron MX-20 X-ray machine. The bones were measured using Fiji ImageJ platform (National Institutes of Health, Bethesda, Maryland, USA; [67]) and one way ANOVA and Student t-test were applied for statistical analysis.

### Histology and immunohistochemistry

Mice were sacrificed at 3 weeks of age, hindlimbs were dissected and fixed in either PFA (histology) or 95% ethanol 5% acetic acid (immunohistochemistry) for 48h in 4^°^C. The limbs were then decalcified in 20% EDTA pH 7.4 over 2 weeks, wax embedded and cut into 6μm sections. Haematoxylin/eosin (H&E) staining was used to visualise the general morphology of the tissue, using the automated Thermo Shandon stainer.

Immunohistochemistry and BrdU labelling (measurement of cell proliferation) were performed as described previously [30]. Primary antibodies were used at a dilution of 1:500 (ER stress: BiP, GRP94 and CRELD2 from R&D Systems; PDIA6 from Abcam, p58IPK from Santa Cruz Biotechnology; ECM: type I collagen, type II collagen from Abcam; matrilin-3 from R&D Systems; type X collagen [68]). BrdU labelled cells were counted using the Watershed algorithm on the Fiji ImageJ platform (National Institutes of Health, Bethesda, Maryland, USA; [67]) and presented as percentage of total cells in the proliferative zone and On-Way ANOVA was used for statistical analysis of data.

### TUNEL assay

TUNEL assay was performed on PFA fixed sections of 3 week old limbs using the Promega Dead-End Fluorimetric Kit as previously [33]. The samples were unmasked using citric buffer boil instead of proteinase K unmasking, which can generate false positives [69]. Positive cells labelled with FITC were counted using the Watershed algorithm on Fiji ImageJ platform (National Institutes of Health, Bethesda, Maryland, USA; [67]) and presented as percentage of all (DAPI stained) cells in selected zones of the growth plate. One-Way ANOVA was used for statistical analysis of the data.

### Isolation of costal chondrocytes

Costal chondrocytes were isolated from pooled litters of 5 day-old mice as described previously [5]. For RNA analysis, the cell pellet was resuspended in TRIzol reagent (Invitrogen), flash frozen and stored at -80^°^C until RNA extraction. For protein analysis, chondrocyte aliquots of 1.5 x 10^5^ cells were re-suspended in SDS loading buffer and frozen at -20^°^C until analysis.

### Microarray analysis of rib chondrocytes

RNA was extracted from isolated chondrocytes using Trizol reagent, according to the manufacturer’s protocol (Life Technologies). Wild type and mutant RNA was pooled from 3 separate extractions, and the submitted to the Genomic Technologies Core Facility, University of Manchester for analysis. RNA integrity was analysed on the 2100 Bioanalyser (Agilent Technologies). A GeneChip 3’ expression assay (Mouse430_2 Affymetrix) was used to analyse gene expression. Quality control checks for control hybridizations were performed using Microarray Suite 5. PPLR is an R package that detects differential gene expression by including probe-level measurement error and calculating the probability of positive log-ratio (PPLR). The differentially expressed (over 1.5 fold change, PPLR of 1.0 and 0.0) genes were subjected to functional annotation analysis using Database for Annotation, Visualisation and Integrated Discovery (DAVID) software [70, 71]. This analysis assigned significantly up/downregulated genes into structural, compartmental and functional-related clusters (GOTERMS). Pathway analysis of the significantly changed genes in the *Xbp1*^Col2CreΔex2^ *Matn3*^V194D^ vs *Xbp1*^WT^ *Matn3*^V194D^ microarray data was performed using the iPathwayGuide platform (Advaita Corp; http://www.advaitabio.com/ipathwayguide). This software analysis tool implements the Impact Analysis approach that takes into consideration the direction and type of all signals on a pathway [72].

### Data availability

The full datasets are available from the NCBI Gene Expression Omnibus (GEO), accession number GSE120308 (http://www.ncbi.nlm.nih.gov/geo/).

### Quantitative RT-PCR analysis of rib chondrocytes

Microarray data were verified by quantitative real time PCR. Briefly, First-strand cDNA was synthesised using random hexamer primers and the GoScript Reverse Transcriptase System (Promega), and qPCR was performed using the SYBR green PCR protocol (Applied Biosystems) on the Chromo4 real-time PCR system (Bio-Rad). Primer sequences are presented in S5 Table. Each experiment included ‘no template’ controls, was run in duplicate and had an 18S RNA control. Each independent experiment was repeated three times, and the results were analysed by independent-samples t-test.

### Western blotting of cartilage homogenates

Tissue homogenates were prepared by homogenising liquid nitrogen frozen 3 week old femoral head tissue in PBS using a microdismembranator for 2 min at 2,000rpm (Sartorius Ltd). Samples (30μg of total protein) were denatured at 95^°^C for 5 min in SDS-PAGE loading buffer containing 100 mM dithiotreitol (DTT. Proteins were separated by SDS-PAGE using Novex NuPAGE 4–12% Bis Tris precast gels in MES running buffer (Fisher Scientific) at 200 V for 60 minutes and electroblotted onto a nitrocellulose membrane for 1 hour at 30 V. Gel loading was assessed using REVERT Total Protein Stain (LI-COR Biosciences) according to manufacturer’s instructions. Membranes were blocked in 3% Milk in PBS-T, incubated with primary antibodies (1:100, ATF4 118155 (Cell Signalling Technology Inc.), ATF6 70B1413.1 (Enzo Life Sciences Inc.), DDIT3 ab11419, IRE1 ab37073, PDIA6 ab154820 (Abcam), PERK C33E10 (Cell Signalling Technology Inc.)) for 1 hour at room temperature, and probed with the appropriate LI-COR IRDye secondary antibody at a concentration of 1:5,000 for 1 hour. Blots were imaged on the LI-COR Odyssey CLx Imaging System. Densitometry quantification was performed using LI-COR proprietary software and verified by ImageJ. The densitometry data was normalised to the total protein stain to account for protein loading. The analysis was undertaken independently by two different researchers, the data was then normalised to wild type protein levels and statistically analysed

## Supporting information

S1 Fig X rays of one year old *Xbp1*^WT^ *Matn3*^V194D^ and *Xbp1*^Col2CreΔex2^ *Matn3*^V194D^ mice showing a striking limb deformity and rotation, potentially due to the pronounced hip dysplasia as well as the constricted bell shaped ribcage characteristic of the *Xbp1*^Col2CreΔex2^ *Matn3*^V194D^ line. (TIF)

S2 Fig Fluorescence imaging of Congo Red staining of mouse growth plates at 3 weeks of age showing potential amyloid-like deposits in the *Xbp1*^WT^ *Matn3*^V194D^ and *Xbp1*^Col2CreΔex2^ *Matn3*^V194D^ cartilage but not rdw cartilage (positive control). Scale bar 100μm.
(TIF)

S3 Fig (A) A heat map generated for top 200 upregulated (in red) and downregulated (in blue) probes in the *Matn3*^V194D^ vs *Matn3*^WT^ (EDM5) analysis showing a high percentage of XBP1-dependent genes (upregulated in the EDM5, downregulated in *Xbp1* null cartilage and in EDM5 lacking Xbp1). (B) Venn diagram analysis showing a comparison of all differentially expressed genes in the compared mouse models. (C) Venn diagram showing differential gene expression in the hypertrophic zone of *Xbp1*^Col2CreΔex2^ vs *Xbp1*^WT^ cartilage compared to the expression profile of the entire growth plate. (D) Venn diagram comparison of the differential gene expression between the MCD and EDM5 mouse models. (E) RT-PCR quantification of the relative levels of *Xbp1*^u^:*Xbp1*^s^ in 5 day old chondrocytes showing higher availability of the *Xbp1*^s^ in the MCDS mouse model compared to the EDM5 mouse (n = 3, Student t-test).
(TIF)

S4 Fig (A) Total protein stain showing protein loading and individual Western blotting for ATF6, IRE1 and PERK on 3 independent biological replicates of whole femoral head cartilage homogenates at 3 weeks of age. B) Total protein stain showing protein loading and individual Western blotting for ATF4, DDIT3, and PDIA6 on 3 independent biological replicates of whole femoral cartilage homogenates at 3 weeks of age. Key: uATF6 –uncleaved ATF6, cATF6 –cleaved (active) ATF6 protein.
(TIF)

S1 Table Significantly upregulated cell proliferation, migration and cellular response to ER stress genes in the *Xbp1*^WT^ *Matn3*^V194D^ vs *Xbp1*^WT^ microarray comparison. (DOCX)

S2 Table List of 57 genes (75 probes) significantly downregulated in the *Xbp1*^WT^ *Matn3*^V194D^ vs *Xbp1*^WT^ comparison and further downregulated in the *Xbp1*^*Col2CreΔex2*^ *Matn3*^*V194D*^ vs *Xbp1*^WT^ *Matn3*^V194D^ comparison. (DOCX)

S3 Table 150 genes significantly upregulated in the *Xbp1*^WT^ *Matn3*^V194D^ vs *Xbp1*^WT^ analysis and downregulated upon removal of Xbp1 (*Xbp1*^*Col2CreΔex2*^ *Matn3*^*V194D*^ vs *Xbp1*^WT^ *Matn3*^V194D^). (DOCX)

S4 Table 79 genes (97 probes) downregulated in *Xbp1*^WT^ *Matn3*^V194D^ vs *Xbp1*^WT^ analysis and upregulated in *Xbp1*^*Col2CreΔex2*^ *Matn3*^*V194D*^ vs *Xbp1*^WT^ *Matn3*^V194D^ dataset. (DOCX)

S5 Table Quantitative RT-PCR primer sequences. (DOCX)
