# Supplementary figures and images for: XBP1 signalling is essential for alleviating mutant protein aggregation in ER-stress related skeletal disease
Source: PLoS Genet. 2019 Jul 1;15(7):e1008215. doi: 10.1371/journal.pgen.1008215 (PMC6625722; doi:10.1371/journal.pgen.1008215)

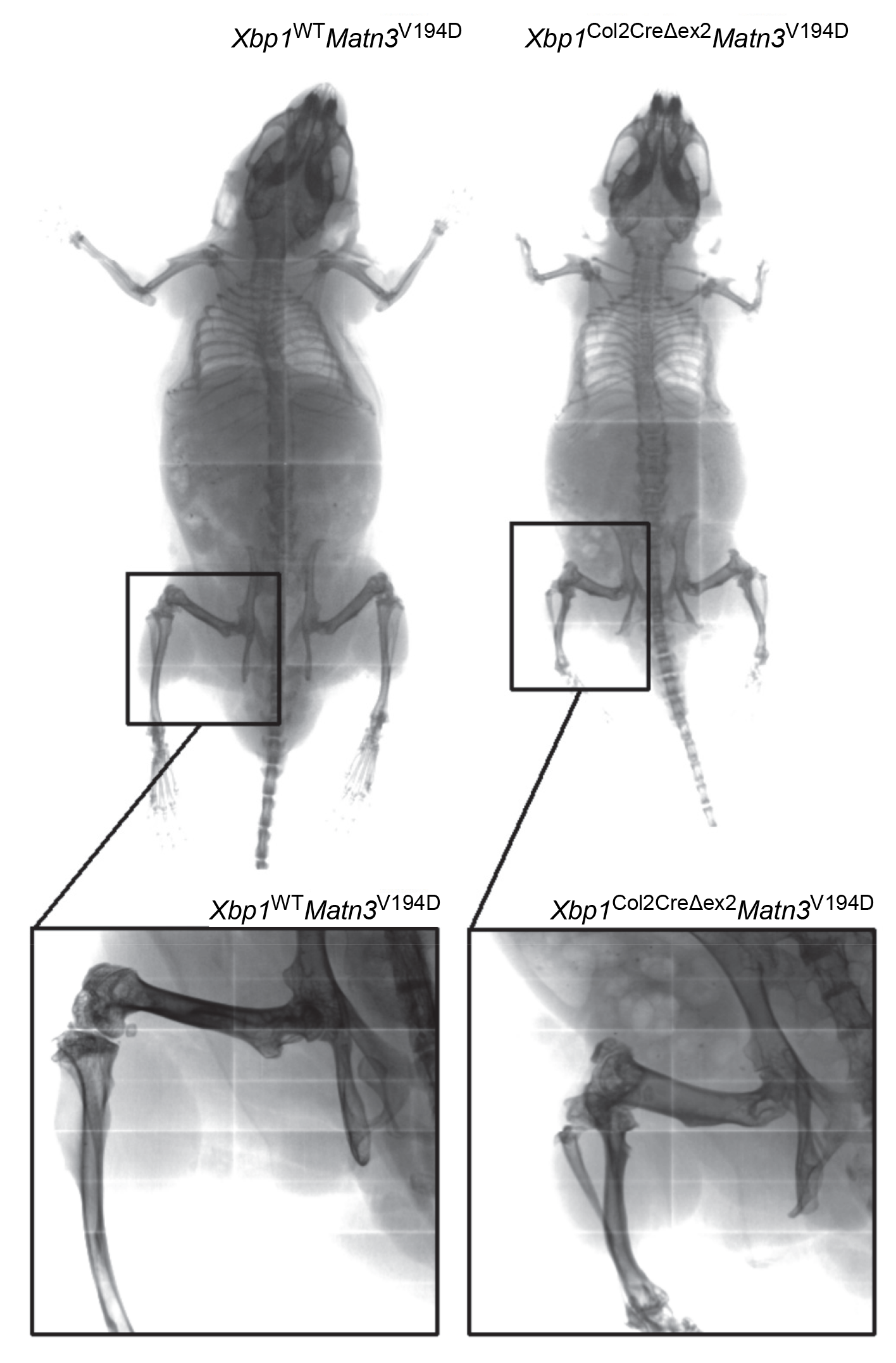

Supplement: S1 Fig — (TIF) [file pgen.1008215.s001.tif]

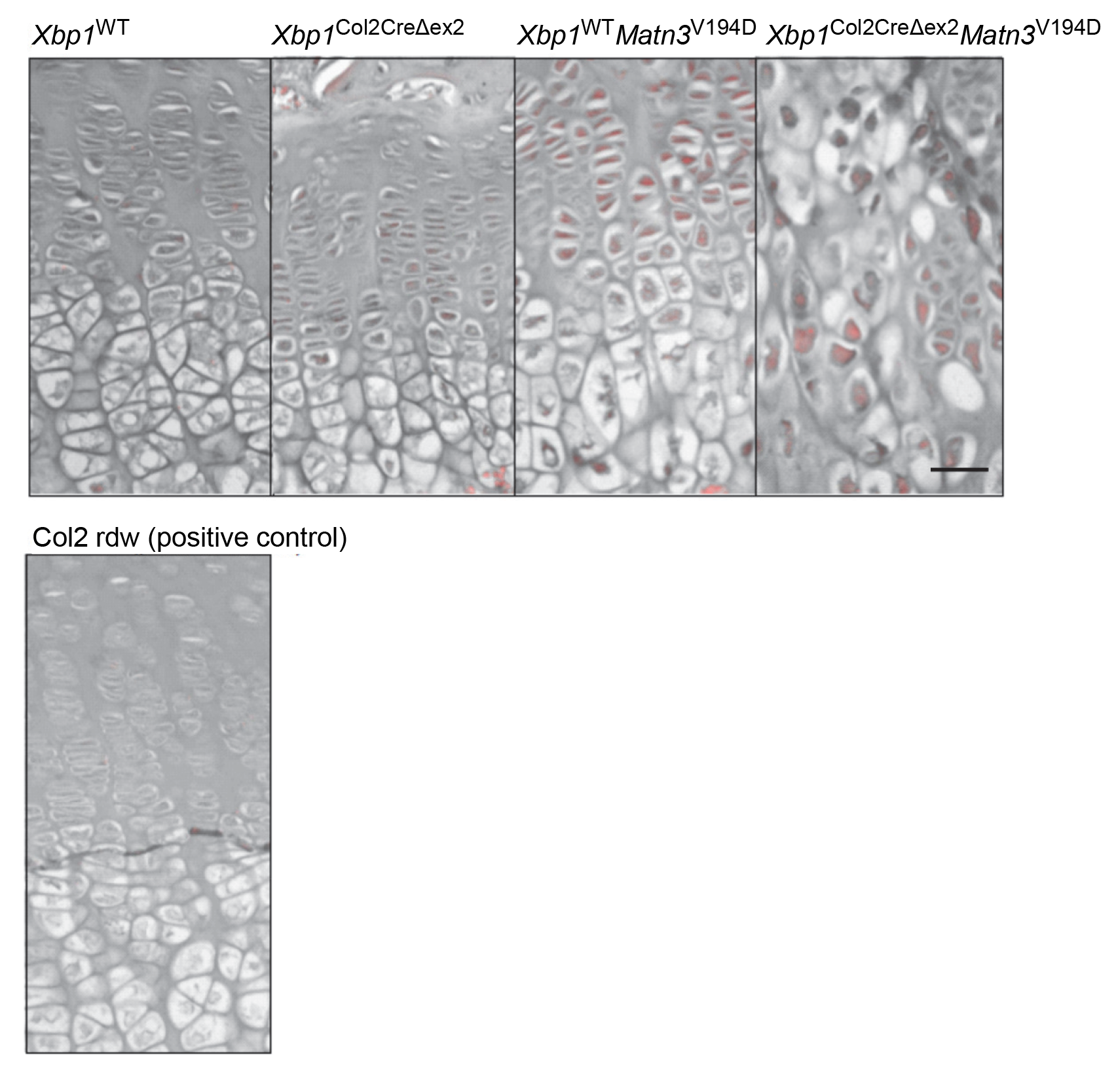

Supplement: S2 Fig — Scale bar 100μm. (TIF) [file pgen.1008215.s002.tif]

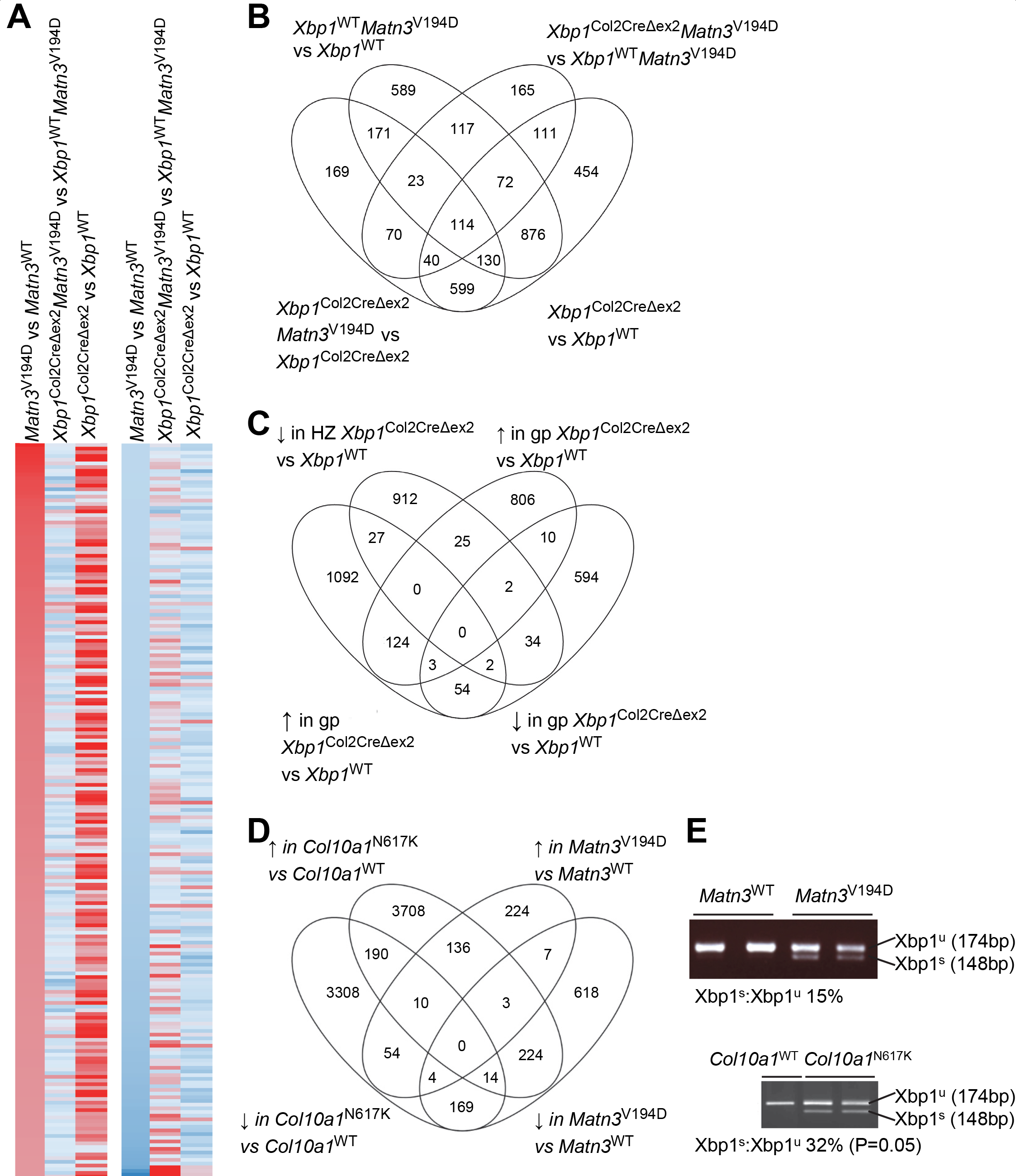

Supplement: S3 Fig — (A) A heat map generated for top 200 upregulated (in red) and downregulated (in blue) probes in the Matn3V194D vs Matn3WT (EDM5) analysis showing a high percentage of XBP1-dependent genes (upregulated in the EDM5, downregulated in Xbp1 null cartilage and in EDM5 lacking Xbp1). (B) Venn diagram analysis showing a comparison of all differentially expressed genes in the compared mouse models. (C) Venn diagram showing differential gene expression in the hypertrophic zone of Xbp1Col2CreΔex2 vs Xbp1WT cartilage compared to the expression profile of the entire growth plate. (D) Venn diagram comparison of the differential gene expression between the MCD and EDM5 mouse models. (E) RT-PCR quantification of the relative levels of Xbp1u:Xbp1s in 5 day old chondrocytes showing higher availability of the Xbp1s in the MCDS mouse model compared to the EDM5 mouse (n = 3, Student t-test). (TIF) [file pgen.1008215.s003.tif]

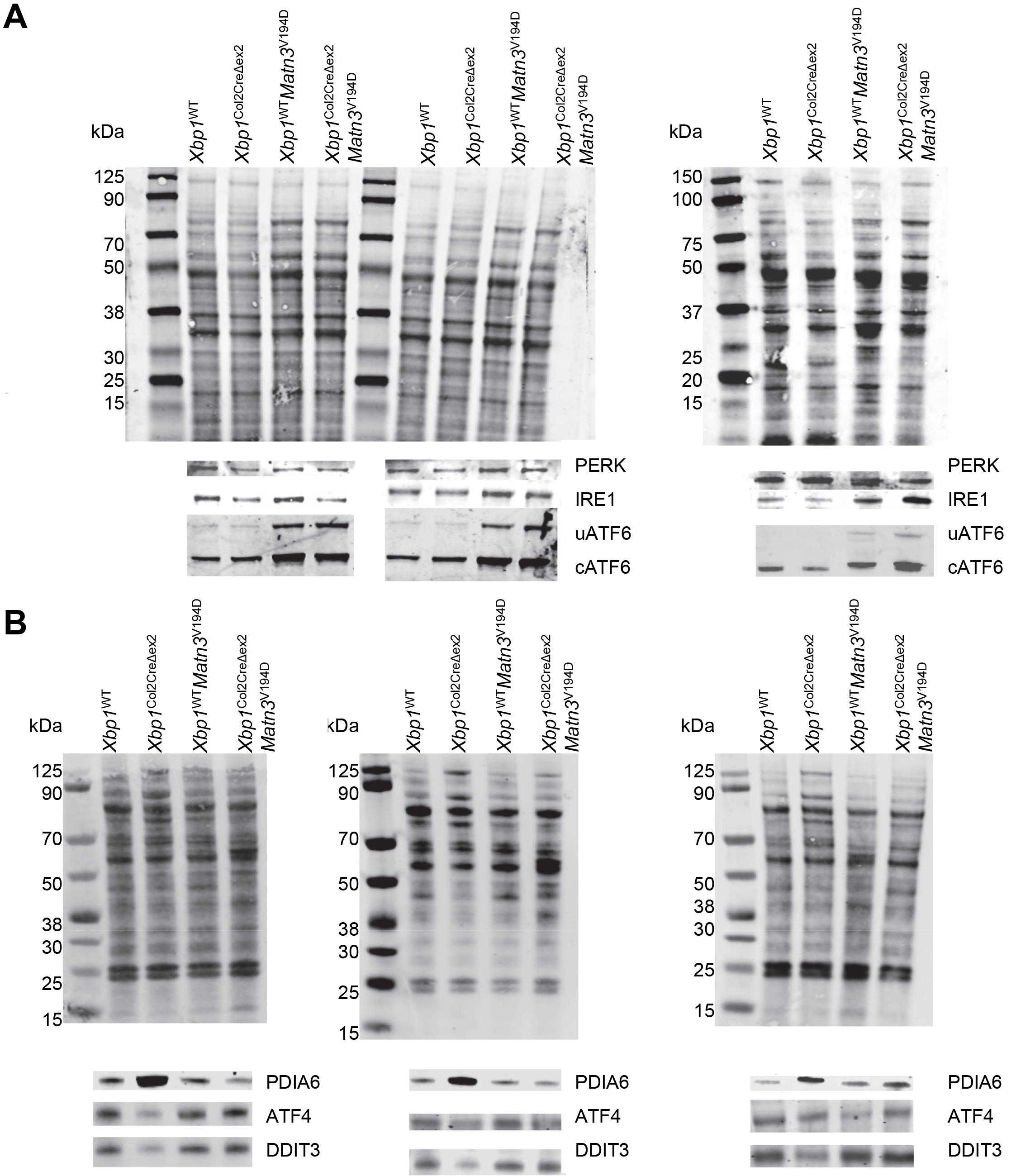

Supplement: S4 Fig — (A) Total protein stain showing protein loading and individual Western blotting for ATF6, IRE1 and PERK on 3 independent biological replicates of whole femoral head cartilage homogenates at 3 weeks of age. B) Total protein stain showing protein loading and individual Western blotting for ATF4, DDIT3, and PDIA6 on 3 independent biological replicates of whole femoral cartilage homogenates at 3 weeks of age. Key: uATF6 –uncleaved ATF6, cATF6 –cleaved (active) ATF6 protein. (TIF) [file pgen.1008215.s004.tif]
