# Supplementary material for: XBP1 signalling is essential for alleviating mutant protein aggregation in ER-stress related skeletal disease
Source: PLoS Genet. 2019 Jul 1;15(7):e1008215. doi: 10.1371/journal.pgen.1008215 (PMC6625722; doi:10.1371/journal.pgen.1008215)
Supplement: S1 Table — (DOCX) [file pgen.1008215.s005.docx]

**S1 Table.** Significantly upregulated cell proliferation, migration and cellular response to ER stress genes in the *Xbp1*^WT^ *Matn3*^V194D^ vs *Xbp1*^WT^ microarray comparison.

| **GO:0030968** | | **GO:0042127** | | **GO:0030335** | |
| --- | --- | --- | --- | --- | --- |
| **endoplasmic reticulum unfolded protein response** | | **regulation of cell proliferation** | | **positive regulation of cell migration** | |
| **Gene symbol** | **Fold change** | **Gene symbol** | **Fold change** | **Gene symbol** | **Fold change** |
| Atf6 | 2.4 | Abl2 | 2.1 | Acvr1 | 2.0 |
| Creld2 | 2.8 | Atf5 | 3.4 | Apc | 4.1 |
| Derl3 | 25.2 | Birc6 | 1.7 | Arhgap5 | 1.9 |
| Dnajc3 | 3.0 | Cdk6 | 1.8 | Ccl5 | 2.7 |
| Ero1l | 23.2 | Csf2 | 3.2 | Cxcl12 | 1.9 |
| Fam129a | 2.3 | Cxcl5 | 4.5 | Epha1 | 13.3 |
| Hsp90b1 | 1.9 | Foxo1 | 1.9 | Ets1 | 2.9 |
| Hyou1 | 6.0 | Fyn | 3.1 | Fam83h | 1.8 |
| Manf | 2.1 | Il4ra | 2.8 | Flt1 | 13.8 |
| Nfe2l2 | 1.6 | Itk | 3.5 | Gcnt2 | 4.8 |
| Pdia3 | 2.3 | Jag1 | 2.3 | Has2 | 3.6 |
| Pdia4 | 2.5 | Mitf | 2.0 | Hbegf | 2.1 |
| Pdia6 | 1.6 | Pla2g4a | 1.8 | Itga6 | 1.8 |
| Ppp1r15b | 1.8 | Plau | 2.4 | Mmp3 | 2.6 |
| Sdf2l1 | 4.7 | Prdm1 | 2.8 | Myo1c | 2.0 |
| Sel1l | 1.8 | Ptgs2 | 2.1 | Pdgfa | 1.7 |
| Stc2 | 2.4 | Tbx19 | 4.7 | Plau | 2.4 |
| Trib3 | 2.2 | Tcfl5 | 1.9 | Prex1 | 1.6 |
| Vapb | 1.8 | Tead1 | 1.7 | Prkca | 3.2 |
| Xbp1 | 3.1 | Tgfb2 | 3.5 | Ptprz1 | 4.0 |
| Yod1 | 1.8 | Tnk2 | 2.3 | Ret | 2.4 |
|  |  | Wnt7a | 14.0 | Sema3e | 2.0 |
|  |  |  |  | Sema7a | 3.0 |
|  |  |  |  | Tgfb2 | 3.5 |
|  |  |  |  | Tnfaip6 | 3.7 |
|  |  |  |  | Vegfa | 2.1 |
