# Supplementary material for: XBP1 signalling is essential for alleviating mutant protein aggregation in ER-stress related skeletal disease
Source: PLoS Genet. 2019 Jul 1;15(7):e1008215. doi: 10.1371/journal.pgen.1008215 (PMC6625722; doi:10.1371/journal.pgen.1008215)
Supplement: S2 Table — (DOCX) [file pgen.1008215.s006.docx]

**S2 Table.** List of 57 genes (75 probes) significantly downregulated in the *Xbp1*^WT^ *Matn3*^V194D^ vs *Xbp1*^WT^ comparison and further downregulated in the *Xbp1^Col2CreΔex2^* *Matn3^V194D^* vs *Xbp1*^WT^ *Matn3*^V194D^ comparison.

| **Gene symbol** | **Fold change *Xbp1*^wt^ *Matn3*^V194D^ vs *Xbp1*^wt^** | **Fold change *Xbp1^Col2CreΔex2^* *Matn*^V194D^ vs *Xbp1*^wt^ *Matn3*^V194D^** | **Gene symbol** | **Fold change *Xbp1*^wt^ *Matn3*^V194D^ vs *Xbp1*^wt^** | **Fold change *Xbp1^Col2CreΔex2^* *Matn3*^V194D^ vs *Xbp1*^wt^ *Matn3*^V194D^** |
| --- | --- | --- | --- | --- | --- |
| Ahsa1 | -1.8 | -2.6 | Mgea5 | -4.2 | -5.3 |
| AI845619 | -2.2 | -2.7 | Mycbp2 | -1.6 | -1.6 |
| Akap13 | -1.5 | -2.1 | Ncor1 | -2.3 | -2.1 |
| Ambra1 | -1.5 | -1.8 | Nr2c2 | -3.6 | -2.1 |
| Birc6 | -1.6 | -1.8 | Peli1 | -1.6 | -3.7 |
| Bptf | -1.5 | -2.0 | Pisd-ps1 | -2.6 | -2.2 |
| Chka | -2.7 | -1.8 | Plagl1 | -1.8 | -1.7 |
| Clcn3 | -1.6 | -1.8 | Prdx6 | -2.6 | -1.9 |
| Copa | -2.9 | -2.1 | Prpf38b | -1.9 | -3.6 |
| Cpsf6 | -1.6 | -1.9 | Psme4 | -2.2 | -2.1 |
| Cryab | -3.5 | -1.9 | Rab14 | -1.9 | -2.3 |
| Cyr61 | -2.9 | -1.8 | Rabl6 | -12.2 | -1.9 |
| Dmtf1 | -2.2 | -2.8 | Rian | -1.7 | -2.3 |
| Dnaja4 | -5.5 | -2.7 | Sc4mol | -2.1 | -2.6 |
| Edil3 | -2.2 | -2.2 | Sgce | -1.7 | -8.7 |
| Ep400 | -1.8 | -2.1 | Slc5a3 | -2.8 | -2.5 |
| Ept1 | -1.7 | -2.1 | Smc2 | -3.3 | -2.0 |
| Fnbp4 | -1.5 | -2.5 | Spred1 | -4.4 | -26.3 |
| Fubp1 | -1.9 | -2.2 | Sqstm1 | -2.7 | -1.9 |
| Gas5 | -2.2 | -2.2 | Tra2a | -2.2 | -2.0 |
| Gnas | -3.3 | -1.8 | Trmt13 | -2.5 | -1.8 |
| Gns | -1.7 | -2.2 | Trmt1l | -1.6 | -1.8 |
| Hipk2 | -1.8 | -1.5 | Trp53bp1 | -1.9 | -1.8 |
| Hist1h2bf | -7.2 | -2.3 | Trpc2 | -3.6 | -2.5 |
| Hist1h4i | -4.1 | -1.9 | Wnk1 | -3.5 | -2.0 |
| Hspa8 | -4.4 | -2.0 | Zcchc7 | -1.6 | -2.6 |
| Hsph1 | -2.0 | -3.3 | Zfp783 | -2.1 | -2.4 |
| Kcnq1ot1 | -1.6 | -2.1 | Zfp942 | -1.5 | -2.9 |
| Map4 | -2.4 | -2.3 |  |  |  |
