# Supplementary material for: XBP1 signalling is essential for alleviating mutant protein aggregation in ER-stress related skeletal disease
Source: PLoS Genet. 2019 Jul 1;15(7):e1008215. doi: 10.1371/journal.pgen.1008215 (PMC6625722; doi:10.1371/journal.pgen.1008215)
Supplement: S3 Table — (DOCX) [file pgen.1008215.s007.docx]

**S3 Table**. 150 genes significantly upregulated in the *Xbp1*^WT^ *Matn3*^V194D^ vs *Xbp1*^WT^ analysis and downregulated upon removal of Xbp1 (*Xbp1^Col2CreΔex2^* *Matn3^V194D^* vs *Xbp1*^WT^ *Matn3*^V194D^).

| **Gene symbol** | **Fold change *Xbp1*^WT^ *Matn3*^V194D^ vs *Xbp1*^WT^** | **Fold change *Xbp1^Col2CreΔex2^* *Matn3*^V194D^ vs *Xbp1*^WT^ *Matn3*^V194D^** | **Gene symbol** | **Fold change *Xbp1*^WT^ *Matn3*^V194D^ vs *Xbp1*^WT^** | **Fold change *Xbp1^Col2CreΔex2^* *Matn3*^V194D^ vs *Xbp1*^WT^ *Matn3*^V194D^** | **Gene symbol** | **Fold change *Xbp1*^WT^ *Matn3*^V194D^ vs *Xbp1*^WT^** | **Fold change *Xbp1^Col2CreΔex2^* *Matn3^V194D^* vs *Xbp1*^WT^ *Matn3*^V194D^** |
| --- | --- | --- | --- | --- | --- | --- | --- | --- |
| Abcb1a | 4.6 | -3.2 | Hyou1 | 6.0 | -3.7 | Rab39b | 2.8 | -2.1 |
| Abhd1 | 4.7 | -1.6 | Il11 | 2.5 | -1.8 | Ranbp2 | 2.7 | -2.1 |
| Abl2 | 2.1 | -2.4 | Il15ra | 3.6 | -1.7 | Rap2b | 2.9 | -1.5 |
| Adam19 | 4.8 | -2.4 | Il1rl1 | 7.9 | -3.7 | Rcan2 | 5.2 | -2.7 |
| Ankrd49 | 2.0 | -1.6 | Il23a | 3.6 | -3.1 | Rcc1 | 2.2 | -1.7 |
| Ankrd6 | 6.8 | -11.3 | Inhba | 2.3 | -2.7 | Rgs2 | 2.2 | -2.8 |
| Birc6 | 1.7 | -1.8 | Itga8 | 16.3 | -1.8 | Rin1 | 19.4 | -16.8 |
| Bmp8a | 7.3 | -4.4 | Itk | 3.5 | -10.7 | Rnasel | 2.0 | -1.6 |
| Bpifc | 10.9 | -2.1 | Kcnn4 | 2.0 | -2.0 | Rps6kb1 | 1.8 | -3.3 |
| Btbd11 | 2.3 | -1.6 | Kdm5c | 2.6 | -2.7 | S100a3 | 1.7 | -2.2 |
| Cav1 | 1.9 | -1.6 | Kdm6b | 3.0 | -1.9 | Scara5 | 3.6 | -2.0 |
| Cbln3 | 7.3 | -2.0 | Klrb1b | 9.2 | -2.7 | Schip1 | 2.8 | -1.5 |
| Ccdc58 | 1.7 | -3.0 | Lifr | 2.6 | -1.9 | Sdf2l1 | 4.7 | -1.6 |
| Ccl5 | 2.7 | -1.8 | Ltbp1 | 16.9 | -30.8 | Sec24a | 2.8 | -2.2 |
| Cd44 | 2.6 | -2.3 | Magt1 | 1.9 | -1.8 | Sema7a | 3.0 | -1.7 |
| Cdh1 | 3.3 | -2.5 | Malt1 | 2.2 | -4.3 | Serpinb5 | 4.1 | -2.5 |
| Cdk5r1 | 2.7 | -1.9 | Map3k4 | 2.0 | -1.6 | Sgcg | 9.1 | -8.2 |
| Chd2 | 1.9 | -1.6 | Mapkapk3 | 2.6 | -1.7 | Sh2d5 | 4.7 | -2.5 |
| Cldn25 | 3.1 | -2.5 | Mark1 | 2.6 | -1.5 | Sipa1l2 | 2.8 | -1.6 |
| Creld2 | 2.8 | -2.3 | Mast4 | 2.3 | -1.8 | Slc11a2 | 8.0 | -2.5 |
| Crisp3 | 15.4 | -9.2 | Med13 | 2.7 | -1.8 | Slc6a14 | 3.0 | -3.7 |
| Cryba4 | 5.0 | -1.9 | Met | 2.4 | -1.5 | Slc7a11 | 5.8 | -1.6 |
| Csf2 | 3.2 | -3.1 | Mfhas1 | 2.4 | -1.9 | Slc7a2 | 2.0 | -2.3 |
| Cxcl5 | 4.5 | -19.0 | Mitf | 2.0 | -1.7 | Slco2a1 | 4.8 | -1.7 |
| Daam1 | 2.4 | -1.6 | Mmp10 | 1.9 | -3.2 | Snapc3 | 3.2 | -3.3 |
| Ddit4l | 10.8 | -2.2 | Mmp3 | 2.6 | -2.8 | Snx29 | 2.1 | -2.3 |
| Defa15 | 4.7 | -1.6 | Msl2 | 2.1 | -1.7 | Sost | 6.3 | -14.8 |
| Dek | 4.6 | -17.3 | Mtmr7 | 1.8 | -1.8 | Spred1 | 3.2 | -26.3 |
| Dnajc3 | 3.0 | -1.5 | Myo10 | 2.9 | -1.9 | Srfbp1 | 1.9 | -1.8 |
| Dph5 | 2.2 | -1.8 | Nfe2l3 | 7.7 | -6.3 | Srrm1 | 1.6 | -3.0 |
| Dst | 1.9 | -2.2 | Ngf | 6.9 | -1.9 | St3gal1 | 2.1 | -1.5 |
| Dus4l | 2.3 | -1.6 | Nipal4 | 12.6 | -10.9 | Stfa2l1 | 1.6 | -2.3 |
| Elovl7 | 3.6 | -3.1 | Noc3l | 2.8 | -1.8 | Tcrb-J | 12.5 | -4.2 |
| Eml1 | 2.4 | -1.6 | Nol8 | 2.6 | -2.5 | Tex18 | 8.5 | -3.0 |
| Ero1lb | 23.2 | -18.7 | Pcdh17 | 4.2 | -1.8 | Tfpi2 | 3.5 | -2.1 |
| Fam13c | 3.0 | -1.6 | Pdlim5 | 2.4 | -1.5 | Tjp2 | 4.2 | -1.6 |
| Fam19a2 | 2.1 | -2.0 | Pex11a | 1.6 | -2.0 | Tmcc3 | 3.4 | -1.5 |
| Fgf17 | 14.8 | -6.9 | Plau | 2.2 | -1.7 | Tmco5 | 14.1 | -3.2 |
| Frmd4a | 3.7 | -1.9 | Plek | 6.3 | -3.3 | Tnrc18 | 1.5 | -2.7 |
| Fus | 1.8 | -2.3 | Pnpla3 | 8.1 | -1.5 | Tpbg | 2.1 | -2.0 |
| Gfra1 | 10.1 | -2.5 | Polr1b | 3.1 | -2.0 | Trio | 1.8 | -1.7 |
| Gjb4 | 3.1 | -1.8 | Pou2af1 | 2.4 | -1.8 | Tspan5 | 1.9 | -1.8 |
| Glis3 | 3.3 | -1.7 | Ppap2b | 2.7 | -1.5 | Tspan8 | 5.8 | -1.6 |
| Gmfb | 2.6 | -2.1 | Ppp1r12b | 2.3 | -2.5 | Ubox5 | 14.9 | -6.3 |
| Gtpbp4 | 3.3 | -1.6 | Prkca | 3.2 | -2.2 | Usmg2 | 15.0 | -5.7 |
| Hao1 | 16.7 | -2.6 | Prl7d1 | 12.0 | -7.2 | Wnt7a | 14.0 | -8.5 |
| Hipk2 | 3.5 | -1.5 | Ptger1 | 4.8 | -2.0 | Xbp1 | 3.1 | -1.6 |
| Hist2h2bb | 11.4 | -7.9 | Ptges | 8.3 | -2.5 | Zdhhc14 | 2.2 | -3.8 |
| Hivep3 | 3.4 | -2.6 | Ptpre | 4.8 | -2.0 | Zfp106 | 2.2 | -1.5 |
| Hmga2 | 3.7 | -1.9 | Pvt1 | 3.3 | -3.1 | Zfp398 | 2.9 | -2.4 |
