# Supplementary material for: XBP1 signalling is essential for alleviating mutant protein aggregation in ER-stress related skeletal disease
Source: PLoS Genet. 2019 Jul 1;15(7):e1008215. doi: 10.1371/journal.pgen.1008215 (PMC6625722; doi:10.1371/journal.pgen.1008215)
Supplement: S4 Table — (DOCX) [file pgen.1008215.s008.docx]

**S4 Table.** 79 genes (97 probes) downregulated in *Xbp1*^WT^ *Matn3*^V194D^ vs *Xbp1*^WT^ analysis and upregulated in *Xbp1^Col2CreΔex2^* *Matn3^V194D^* vs *Xbp1*^WT^ *Matn3*^V194D^ dataset.

| **Gene symbol** | **Fold change *Xbp1*^WT^ *Matn3*^V194D^ vs *Xbp1*^WT^** | **Fold change *Xbp1^Col2CreΔex2^* *Matn3*^V194D^ vs *Xbp1*^WT^ *Matn3*^V194D^** | **Gene symbol** | **Fold change *Xbp1*^WT^ *Matn3*^V194D^ vs *Xbp1*^WT^** | **Fold change *Xbp1^Col2CreΔex2^* *Matn3*^V194D^ vs *Xbp1*^WT^ *Matn3*^V194D^** | **Gene symbol** | **Fold change *Xbp1*^WT^ *Matn3*^V194D^ vs *Xbp1*^WT^** | **Fold change *Xbp1^Col2CreΔex2^* *Matn3*^V194D^ vs *Xbp1*^WT^ *Matn3*^V194D^** |
| --- | --- | --- | --- | --- | --- | --- | --- | --- |
| Abhd3 | -2.2 | 1.8 | Igfbp5 | -2.2 | 2.7 | Snurf | -2.5 | 3.2 |
| Acta1 | -2.1 | 2.8 | Kera | -2.0 | 3.4 | St3gal5 | -2.4 | 2.1 |
| Adh1 | -2.8 | 2.8 | Khdrbs3 | -2.0 | 2.1 | Tceal3 | -2.7 | 1.8 |
| Adm | -7.2 | 2.3 | Lgals1 | -2.0 | 2.3 | Tecr | -2.5 | 2.2 |
| Ankmy2 | -2.9 | 2.3 | Ly6e | -2.2 | 2.4 | Timp4 | -2.3 | 3.8 |
| Ankrd29 | -3.1 | 2.0 | Maob | -2.8 | 2.6 | Tmem141 | -4.7 | 3.5 |
| Anp32a | -1.9 | 10.3 | Map7d2 | -83.0 | 3.2 | Tmem173 | -2.4 | 2.5 |
| Aspn | -1.9 | 1.9 | Mbd3 | -2.1 | 2.0 | Tnc | -1.9 | 1.7 |
| Car8 | -3.2 | 2.4 | Mdfi | -5.3 | 4.8 | Tox3 | -1.8 | 2.3 |
| Cby1 | -3.2 | 2.8 | Mfap2 | -2.6 | 2.3 | Tubb5 | -3.4 | 2.2 |
| Cdh11 | -2.2 | 2.5 | Mfap4 | -1.9 | 2.5 | Ube2d1 | -2.7 | 2.0 |
| Cebpd | -2.4 | 2.0 | Mlf2 | -3.3 | 2.2 | Ulk1 | -6.5 | 6.6 |
| Col1a1 | -2.7 | 2.1 | Mt1 | -7.4 | 2.3 | Zic1 | -2.2 | 2.6 |
| Ddx41 | -3.0 | 1.8 | Ndnf | -1.9 | 1.9 |  |  |  |
| Dmc1 | -2.1 | 2.1 | Nt5dc2 | -2.7 | 2.2 |  |  |  |
| Dnajc22 | -1.7 | 1.7 | Olfml2b | -2.0 | 2.6 |  |  |  |
| Dusp15 | -2.6 | 2.5 | Pik3ip1 | -4.9 | 3.2 |  |  |  |
| Edn2 | -2.5 | 2.2 | Plin2 | -3.1 | 2.0 |  |  |  |
| Fabp5 | -1.9 | 2.0 | Ppp1r1b | -3.0 | 2.6 |  |  |  |
| Fam109b | -5.3 | 3.6 | Pstpip1 | -2.0 | 2.3 |  |  |  |
| Fam89b | -2.1 | 2.1 | Ptn | -3.3 | 2.3 |  |  |  |
| Foxa1 | -3.0 | 3.8 | Rad51c | -3.0 | 2.0 |  |  |  |
| Foxo3 | -7.6 | 4.1 | Rassf5 | -2.0 | 2.3 |  |  |  |
| Fut4 | -2.1 | 2.2 | Rnd3 | -2.3 | 2.5 |  |  |  |
| Gadd45g | -2.5 | 2.3 | Serhl | -6.6 | 3.8 |  |  |  |
| Gnb1l | -2.6 | 2.0 | Serpina1a | -6.6 | 2.5 |  |  |  |
| Guk1 | -2.8 | 2.3 | Serpina1b | -16.2 | 5.5 |  |  |  |
| Hist1h4a | -6.5 | 1.9 | Sfn | -2.5 | 2.4 |  |  |  |
| Hoxc5 | -2.5 | 2.0 | Sfrp2 | -4.0 | 2.1 |  |  |  |
| Ibsp | -10.2 | 5.6 | Shisa3 | -3.8 | 3.6 |  |  |  |
| Ier5l | -2.6 | 1.6 | Sigmar1 | -1.8 | 1.9 |  |  |  |
| Ift27 | -2.6 | 2.2 | Slc10a6 | -3.2 | 6.0 |  |  |  |
| Igf1 | -2.2 | 3.6 | Snai1 | -2.5 | 2.4 |  |  |  |
