# Supplementary material for: XBP1 signalling is essential for alleviating mutant protein aggregation in ER-stress related skeletal disease
Source: PLoS Genet. 2019 Jul 1;15(7):e1008215. doi: 10.1371/journal.pgen.1008215 (PMC6625722; doi:10.1371/journal.pgen.1008215)
Supplement: S5 Table — (DOCX) [file pgen.1008215.s009.docx]

**S5 Table.** Quantitative RT-PCR primer sequences.

| **Gene symbol** | **Primers** |
| --- | --- |
| *Atf6* | F 5’-TAC CAC CCA CAA CAA GAC CA-3’  R 5’-TGA TGA TCC CGG AGA TAA GG-3’ |
| *Creld2* | F 5’-TCA GTG AGA TCC GGC TTC TG-3’  R 5’-GAA GAC AGC ACG CTT TCA GT-3’ |
| *Ddit3* | F 5’-ATG ATG GCT TGG CCA GTG-3’  R 5’-CCA TTT TCT CCA ACA TCC AAT C-3’ |
| *Derl1* | F 5’- CTG TCC ACA CCT CAG TTT TTG TA-3’  R 5’-GGC ACA CCA AAT CCT GAC A-3’ |
| *Grp94* | F 5’-GCA CCA TGA GGG TCC TGT-3’  R 5’-CAT CAT CAG CTC TGA CGA ACC-3’ |
| *Ire1* | F 5’-CTG CCT CCA GCT ACC AAG A-3’  R 5’-TCC CCA CAT ACA GTG TCA TCA-3’ |
| *Manf* | F 5’-GAC AGC CAG ATC TGT GAA CTA AAA-3’  R 5’-TTT CAC CCG GAG CTT CTT C-3’ |
| *Perk* | F 5’-CCT TGG TTT CAT CTA GCC TCA-3’  R 5’-ATC CAG GGA GGG GAT GAT-3’ |
| *Pdia6* | F 5’-TGG TTC CTT TCC TAC CAT CAC T-3’  R 5’-ACT TTC ACT GCT GGA AAA CTG C-3’ |
